# Protective and Detoxifying Enzyme Activity and *ABCG* Subfamily Gene Expression in *Sogatella furcifera* Under Insecticide Stress

**DOI:** 10.3389/fphys.2018.01890

**Published:** 2019-01-08

**Authors:** Cao Zhou, Hong Yang, Zhao Wang, Gui-Yun Long, Dao-Chao Jin

**Affiliations:** ^1^Provincial Key Laboratory for Agricultural Pest Management of Mountainous Regions, Institute of Entomology, Guizhou University, Guiyang, China; ^2^College of Tobacco Science of Guizhou University, Guiyang, China; ^3^College of Environment and Life Sciences, Kaili University, Kaili, China

**Keywords:** white-backed planthopper, detoxifying enzyme, protective enzyme, ATP-binding cassette transporter, insecticide stress, response mechanism

## Abstract

*Sogatella furcifera*, an important migratory pest of rice, has substantial detrimental effects on rice production. To clarify the mechanism whereby *S. furcifera* responds to insecticide stress, we measured the activity of its protective [superoxide dismutase (SOD); peroxidase (POD); catalase (CAT)] and detoxifying [carboxylesterase (CarE); glutathione *S*-transferase (GST); mixed-function oxidase (MFO)] enzymes and the expression levels of its ATP-binding cassette subfamily G (*ABCG*) transporter genes in response to sublethal concentrations (LC_10_ and LC_25_) of the insecticides thiamethoxam, buprofezin, and abamectin. On the bases of the transcriptome data and the *ABCG* genes of *Laodelphax striatellus*, we obtained 14 full-length *ABCG* sequences for *S. furcifera*. RT-qPCR results showed that 13, 12, and 9 *sfABCG* genes were upregulated in the presence of thiamethoxam, buprofezin, and abamectin, respectively, at LC_10_. Moreover, 13 and 7 *sfABCG* genes were upregulated following treatment with thiamethoxam and abamectin, respectively, at LC_25_. Enzyme activity assays showed that although thiamethoxam, buprofezin, and abamectin induced GST, CarE, CAT, POD, and SOD activity, they did so at different concentrations and exposure times. The activity of MFO was generally inhibited with prolonged exposure to the three insecticides, with the inhibitory effect being most significant at 72 h. These results indicate that *S. furcifera* differs in its response to different types or concentrations of insecticides. Taken together, our results lay the foundations for gaining a deeper understanding of the mechanisms underlying the adaptation of *S. furcifera* to different types of insecticides, which would be of considerable significance for the development of effective pest management strategies.

## Introduction

*Sogatella furcifera*, an important pest of rice, causes serious problems in rice production by sucking phloem sap from the rice plant, inflicting damage through oviposition, and transmitting viral diseases (Zhou et al., 2008). Although the use of insecticides has traditionally been an important means of control for this rice pest (Endo and Tsurumachi, 2001; Nizamani et al., 2002), recent research has shown that sublethal concentrations of insecticides can affect the reproduction, development, and chemical susceptibility of insects in such a way that it could potentially result in the resurgence of pests (Zhou et al., 2017).

In general, the detoxification process in insects can be divided into three phases: phase I, phase II (involving metabolizing enzymes), and phase III (involving transporters) (Xu et al., 2005). The main enzymes involved in the phase I and phase II detoxification processes are P450 monooxygenase, glutathione *S*-transferase (GST), and carboxylesterase (CarE) (Xiao et al., 2018), whereas the ATP-binding cassette (ABC) transporters are the main components of phase III (Ferreira et al., 2014). In this regard, it has previously been observed that when the nymphs of *Locusta migratoria* were treated with chlorantraniliprole at LC_50_, only the activities of esterase (EST) and GST increased on the first day of treatment, whereas mixed-function oxidase (MFO) activity increased only at 3 days after treatment (Cao et al., 2017). In addition, superoxide dismutase (SOD), peroxidase (POD), and catalase (CAT) are three important protective enzymes in insects that play roles in immunity, preventing free-radical-associated damage, and protecting cells from adverse environmental effects (Dubovskiy et al., 2008; Bi et al., 2010). It has previously been reported that in response to treatment with abamectin at LC_10_ and LC_20_ for 12 h, the activities of SOD, POD, and CAT in *Harmonia axyridis* were higher relative to those in the untreated control group, although these activities gradually returned to normal levels as time progressed (Yang et al., 2015). Furthermore, in response to treatment with imidacloprid at LC_10_ and LC_20_, the SOD and POD activities in *Aphidius gifuensis* initially appeared to be inhibited but were subsequently stimulated, with the highest activity occurring at 36 h. Moreover, it has been found that with an increase in insecticide concentration, SOD, POD, and CAT activities show a decreasing trend (Zhu et al., 2015).

The ABC transporters comprise a large family of proteins that mediate the transport of inorganic ions, sugars, amino acids, lipids, lipopolysaccharides, peptides, metals, xenobiotics, and chemotherapeutic drugs (Higgins, 1992). In insects, this family can be subdivided into eight major subfamilies (A–H) (Dean et al., 2001). Studies of the ABC transporters in eukaryotes have revealed that they are capable of transporting structurally unrelated compounds (Dassa and Bouige, 2001; Dean et al., 2001), and researchers are thus increasingly focusing on the roles of these proteins in the transport of exogenous substances and in insecticide resistance in insects. Recent studies have shown that the expression of ABC transporters is directly related to the development of insecticide resistance (Silva et al., 2012a; Dermauw and Van Leeuwen, 2014). After treatment of *Bactrocera dorsalis* with malathion, abamectin, and beta-cypermethrin at an LD_50_ concentration, 4, 10, and 14 *bdABC* genes were significantly upregulated, respectively (Xiao et al., 2018). Quantitative polymerase chain reaction (qPCR) analysis has revealed that eight ABC transporters in the ABCB/C/D/G subfamilies were upregulated in strains of *Laodelphax striatellus* resistant to chlorpyrifos, deltamethrin, and imidacloprid, compared with those in a susceptible strain (Sun et al., 2017). In *Plutella xylostella*, RNA sequencing (RNA-seq) analysis showed that ABC transporters from the ABCA/C/G/H/F subfamilies were overexpressed in chlorpyrifos-resistant strains (You et al., 2013). Nevertheless, despite the insights gained from these studies, our current understanding of the role of ABC transporters in insect resistance to insecticides remains limited.

At present, little is known regarding the effects of insecticides on the activities of the detoxifying and protective enzymes and ABC transporters of *S. furcifera*. Accordingly, in this study, we sought to gain insights into the roles of these enzymes and the *sfABCG* subfamily genes in the response of *S. furcifera* to insecticide-induced stress. To this end, we exposed this insect to sublethal concentrations of three insecticides (thiamethoxam, abamectin, and buprofezin) and subsequently monitored the changes in enzyme activity and gene expression levels.

## Materials and Methods

### Insects and Insecticides

In 2013, *S. furcifera* individuals were collected from a rice field in Huaxi, Guiyang, Guizhou, China (26°31.302″ N, 106°62.294″ E) and maintained on rice seedlings in the laboratory at 25 ± 1°C and 70 ± 10% relative humidity under a 16:8 h (light:dark) photoperiod, without exposure to insecticides. For the purposes of this study, we used third-instar nymphs. Thiamethoxam (96%: technical formulation) was obtained from PFchem, Co., Ltd. (Nanjing, China); abamectin (96.4%: technical formulation) was obtained from Shandong Qilu King-Phar Pharmaceutical, Co., Ltd. (Shandong, China); and buprofezin (97%: technical formulation) was obtained from the Guangxi Pingle Pesticide Factory (Guangxi, China).

### Insect Treatments and Sample Collection

For the insecticide treatments, we used the rice stem dipping method (Zhou et al., 2017). Three 100 third-instar nymphs were transferred to and reared separately in glass tubes (300 mm high × 30 mm diameter) that were open at both ends and contained rice seedlings dipped in a sublethal concentration (LC_10_ or LC_25_) of thiamethoxam, abamectin, or buprofezin. Rice stems treated with distilled water were used as a control. The insects exposed to each treatment were maintained at 25 ± 1°C and 70 ± 10% relative humidity under a 16:8 h (light:dark) photoperiod in an artificial climate box. After 48 h, 15 surviving insects from each treatment were randomly collected for extraction of RNA for a quantitative reverse-transcription PCR (RT-qPCR) assay. In addition, samples were taken at 6, 12, 24, 48, and 72 h after the treatment to determine the activity of the target enzymes. The LC_10_ and LC_25_ values (Supplementary Table S1) of thiamethoxam, abamectin, and buprofezin for *S. furcifera* were based on previously presented results (Liu et al., 2015).

### Gene Identification

The RNA-seq transcriptome database of *S. furcifera* was sequenced and annotated as described previously (Zhou et al., 2018). With the reported *ABCG* gene of *L. striatellus* as a reference, Geneious R9 software (Kearse et al., 2012) was used to assemble the transcriptome data to obtain the corresponding sequences for *S. furcifera*. In addition, each of the putative *ABCG* sequences was used as a query to search the NCBI protein database^1^ to further validate their identity.

### Sequence Verification

Specific primers were designed and used to amplify the internal cDNA fragments. PCRs were carried out using Sangon Biotech (Shanghai, China) Taq polymerase, under the following conditions: initial denaturation at 94°C for 3 min; 30 cycles of denaturation at 94°C for 30 s, annealing at 55–60°C for 30 s, and elongation at 72°C for 1–2 min; with a final elongation at 72°C for 10 min. Specific primers for amplification of the 3′ and 5′ ends were designed using Primer Premier 6.0 (Premier Biosoft International, Palo Alto, CA, United States). Using a SMARTer^®^ RACE 5′/3′ Kit (Clontech, Mountain View, CA, United States), 3′ and 5′ rapid amplification of cDNA ends (RACE) were performed. Total RNA was extracted from 10 fifth-instar nymphs according to the instructions of an HP Total RNA Kit (Omega Bio-Tek, Norcross, GA, United States). Synthesis of the first-strand cDNA and PCR amplifications were carried out according to the instructions of a SMARTer^®^ RACE 5′/3′ Kit. SeqAmp DNA Polymerase (a SMARTer^®^ RACE 5′/3′ Kit component) was used for the RACE PCR, under the following conditions: 25 cycles of 94°C for 30 s, 60–70°C (depending on the primer) for 30 s, and 72°C for 3 min. The overlapping PCR products were purified using an E.Z.N.A^®^ Gel Extraction Kit, cloned into a linearized pRACE vector (a SMARTer^®^ RACE 5′/3′ Kit component), and sequenced by Sangon Biotech (Shanghai, China). The RACE sequences were assembled on the basis of the partial cDNA sequences corresponding to each fragment.

### Sequence Alignment and Phylogenetic Analysis

Using ORF finder^2^, we identified the open reading frames (ORFs) of the *sfABCG* genes and determined the amino acid sequences of the encoded proteins. The Pfam program^3^ and a search of the NCBI Conserved Domain Database^4^ were used to identify the conserved domains (nucleotide-binding and transmembrane domains) of all putative *ABCG* genes. The *ABCG* gene sequences were then subjected to phylogenetic analysis, using the neighbor-joining method and a bootstrap test with 1,000 replicates in the MEGA program package, v. 6.0 (Tamura et al., 2011).

### Gene Expression Analysis

The mRNA levels of the ABC transporter genes under different insecticide treatments were measured by RT-qPCR using FastStart Essential DNA Green Master Mix (Roche, Indianapolis, IN, United States) in a CFX96^TM^ real-time quantitative PCR system (BioRad, Hercules, CA, United States). Total RNA was extracted as described above and quantified using a NanoDrop 2000 spectrophotometer (Thermo Fisher Scientific, Waltham, MA, United States) according to the manufacturer’s protocols. The RNA concentration was adjusted to 0.8 μg/μL with diethyl pyrocarbonate-treated H_2_O, and 0.8 μg of RNA was then reverse transcribed in a 20-μL reaction volume, using the PrimeScript RT Reagent Kit and gDNA Eraser (TaKaRa, Shiga, Japan), with ribosomal protein L9 (GenBank Accession No. KM885285) as an internal control. Specific primer pairs for each gene were designed using Primer Premier 6 (Supplementary Table S2). Each RT-qPCR was conducted in a 20-μL mixture containing 1 μL of sample cDNA, 1 μL of each primer (10 μM), 7 μL of diethyl pyrocarbonate-treated H_2_O, and 10 μL of FastStart Essential DNA Green Master Mix. The qPCR cycling parameters were as follows: 95°C for 10 min, followed by 40 cycles of 95°C for 30 s and 60°C for 30 s. Melting curve generation was performed from 65 to 95°C. To check the reproducibility of the assay results, the qPCR for each sample was performed using three technical replicates and three biological replicates. The comparative 2^-ΔΔCT^ method (Livak and Schmittgen, 2001) was used to calculate the relative quantification.

### Enzyme Activity Assay

In this study, we performed the following enzyme activity assays: the nitroblue tetrazolium reduction method for SOD; the guaiacol method for POD; a spectrophotometric method for CAT (based on the ultraviolet absorption of peroxide released from the activity of CAT on hydrogen peroxide); a colorimetric method for GST (based on the GST-catalyzed reaction between glutathione and 1-chloro-2,4-dinitrobenzene); and a colorimetric method for CarE (based on the CarE-catalyzed transformation of 1-naphthyl acetate to naphthyl ester, which then reacts with the Fast Blue RR salt to form an azo dye). These assays were conducted using respective commercial assay kits (Comin Biotechnology, Co., Ltd., Suzhou, China). MFO activity was measured according to the method reported by Qian et al. (2008). To check the reproducibility of the results, the enzyme activity assays for each insecticide treatment were performed using four biological replicates.

### Statistical Analyses

All data were analyzed using Bonferroni corrections for multiple comparisons when the variance was homogeneous. When the variance was non-homogeneous, the Wilcoxon signed-rank test was used. In addition, the Kruskal–Wallis test was used to verify the temporal shifts within the effects of the same insecticide. All analyses were performed using SPSS version 22.0 (SPSS, Chicago, IL, United States) and the data are presented as the mean ± standard error (SE) of three or four biological replicates.

## Results

### Identification and Characterization of ABC Subfamily G Transporter Genes

Using the reported *ABCG* gene of *L. striatellus* as a reference, Geneious R9 software was used to assemble the transcriptome data to obtain the corresponding sequences for *S. furcifera*. We verified 14 *sfABCG* genes by RT-qPCR and RACE (Supplementary Table S3). The designation, accession number, length, ORF size, theoretical isoelectric point, and molecular weight of all the *sfABCG* genes are summarized in Table 1. The ORFs of all gene sequences ranged from 603 to 967 bp. We initially identified the characteristic nucleotide-binding domains of ABC transporters using Pfam. The nucleotide-binding and transmembrane domains of all genes were similar to those of *L. striatellus* (Supplementary Figure S2). As determined from the neighbor-joining tree generated from phylogenetic analysis of the *ABCG* genes of *S. furcifera, L. striatellus, Tribolium castaneum*, and *B. dorsalis*, the corresponding genes of each subfamily are clustered together (Supplementary Figure S1).

**Table 1:** Full-length ATP-binding cassette subfamily G (*ABCG*) transporter genes identified from *Sogatella furcifera.*

Gene name	Accession number	Product size (bp)	Number of coded amino acids (aa)	Molecular weight	Theoretical pI
*sfABCG1*	MH481837	2139	632	71358.37	8.66
*sfABCG2*	MH481838	2072	686	76136.25	9.08
*sfABCG3*	MH481839	2140	663	74617.45	7.84
*sfABCG4*	MH481840	1998	607	69437.43	8.94
*sfABCG5*	MH481841	3242	967	106143.85	9.27
*sfABCG6*	MH481842	2146	615	69247.55	8.83
*sfABCG7*	MH481843	2224	710	79663.87	7.49
*sfABCG8*	MH481844	2392	631	71421.90	8.64
*sfABCG9*	MH481845	1871	617	69360.48	9.07
*sfABCG10*	MH481846	2308	642	71283.78	9.16
*sfABCG11*	MH481847	1961	603	68077.09	8.85
*sfABCG12*	MH481848	1865	620	70004.86	8.15
*sfABCG13*	MH481849	2187	722	82286.16	7.86
*sfABCG14*	MH481850	2186	720	79754.97	7.83

### Effect of Insecticide Treatment on *sfABCG* Gene Expression

**FIGURE 1:**
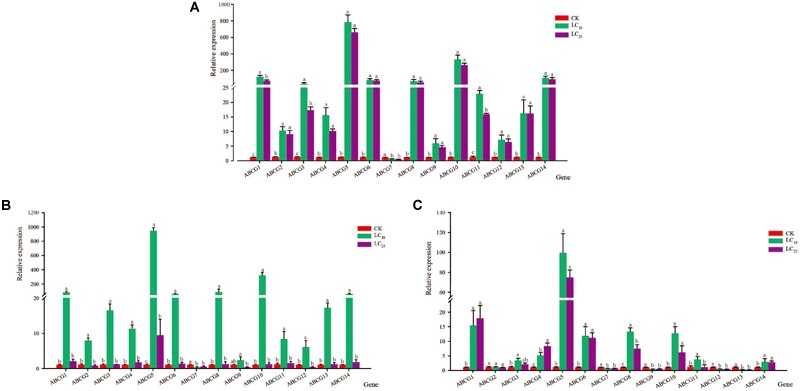
Relative expression levels of 14 putative ABC subfamily G (*ABCG*) transporter genes in *Sogatella furcifera* under treatment with sublethal concentrations (LC_10_ and LC_25_) of thiamethoxam, buprofezin, and abamectin. The mean value ± SE was used to analyze the relative expression levels under different insecticide concentrations using the ΔΔCt method, with non-insecticide treatment (CK) as a reference. Different letters indicate significant differences among treatments at the same time. **(A)** Thiamethoxam; **(B)** buprofezin; and **(C)** abamectin.

After exposing third-instar nymphs of *S. furcifera* to different concentrations of thiamethoxam for 48 h, we examined the relative expression levels of the 14 *sfABCG* genes. The results showed that the expression of only *sfABCG7* was significantly downregulated (2.4-fold) after treatment with the insecticide at LC_10_ (Figure 1), whereas the other 13 *sfABCG* genes were significantly upregulated. Among these 13 genes, *sfABCG5* (766.6-fold) and *sfABCG9* (5.8-fold) showed the highest and lowest upregulation, respectively. Responses to the LC_25_ treatment were similar to those observed for the LC_10_ treatment, with only *sfABCG7* being significantly downregulated (3.5-fold) and the remaining 13 genes being significantly upregulated by 4.5- to 643.8-fold. However, we found that the expression levels of the upregulated genes showed a decreasing trend with increasing insecticide concentration, with *sfABCG3* showing significantly different expression levels in response to the LC_10_ and LC_25_ treatments (Wilcoxon signed-rank test *P* < 0.05).

After treatment with buprofezin at LC_10_, the relative expression levels of the 14 *sfABCG* genes showed trends similar to those observed for thiamethoxam at LC_10_, with only the *sfABCG7* gene being significantly downregulated (3.0-fold), and the other 13 genes all being upregulated (significantly in the case of 12), from 6.0- to 924.0-fold. Although the expression of *sfABCG9* was upregulated relative to that in the control, the difference was not significant (Figure 1). Buprofezin treatment at LC_25_ resulted in a significant upregulation of *sfABCG5* relative to the control, whereas *sfABCG7* was significantly downregulated by 2.2-fold (Bonferroni-corrected *P =* 0.02) compared with the control level.

After treatment with abamectin at LC_10_, nine *sfABCG* genes (*sfABCG1, sfABCG3, sfABCG4, sfABCG5, sfABCG6, sfABCG8, sfABCG10, sfABCG11*, and *sfABCG14*) were significantly upregulated in the range of 3.2- to 97.4-fold (Figure 1). In contrast, compared with the control levels, the expression levels of *sfABCG7, sfABCG9, sfABCG12*, and *sfABCG13* were significantly downregulated in response to abamectin treatment at LC_10_ and LC_25_ concentrations, with *sfABCG13* being the most downregulated by 6.0-fold (LC_10_) and 13.3-fold (LC_25_). In response to abamectin exposure at the LC_25_ concentration, *sfABCG1, sfABCG4, sfABCG5, sfABCG6, sfABCG8*, and *sfABCG14* were significantly upregulated by 17.1-, 8.1-, 73.1-, 10.98-, 7.4-, and 2.7-fold, respectively, compared with the control levels. Interestingly, the expression levels of both *sfABCG1* and *sfABCG4* were upregulated with increasing abamectin concentration, with the difference being significant in the case of *sfABCG4* (Bonferroni-corrected *P =* 0.04; Figure 1).

**FIGURE 2:**
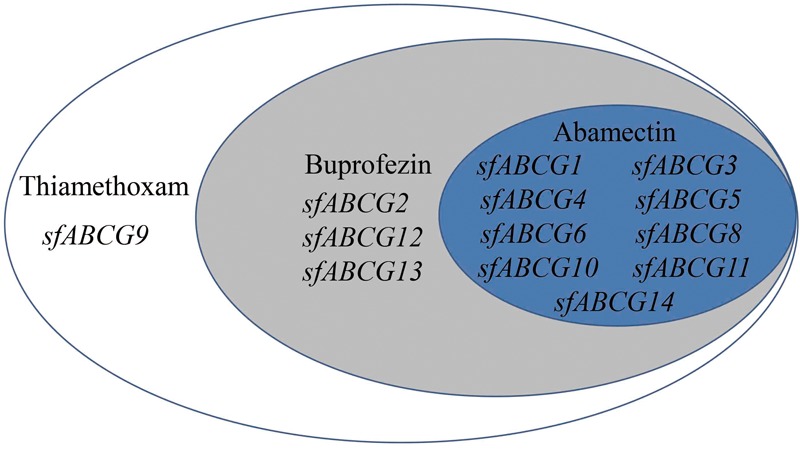
Summary of the significantly upregulated genes in *Sogatella furcifera* under treatment with the insecticides thiamethoxam, buprofezin, and abamectin. The Venn diagram shows the putative ABC subfamily G (*ABCG*) transporter genes found to be significantly upregulated in the insecticide-treated insects compared with the untreated controls.

To gain a more intuitive understanding of the gene upregulation pattern in response to insecticide exposure, a Venn diagram was generated for the 13 significantly upregulated *sfABCG* genes after treatment with the three insecticides at LC_10_ (Figure 2). Among these, *sfABCG9* was upregulated by thiamethoxam only; *sfABCG2, sfABCG12*, and *sfABCG13* were upregulated by thiamethoxam and buprofezin; and *sfABCG1, sfABCG3, sfABCG4, sfABCG5, sfABCG6, sfABCG8, sfABCG10, sfABCG11*, and *sfABCG14* were upregulated by all three insecticides. Among the latter group, the *sfABCG5* gene showed the highest upregulation responses, with expression levels 766.6-, 924.0-, and 97.4-fold higher than those of the control in response to thiamethoxam, buprofezin, and abamectin treatments, respectively.

### Activity of Detoxifying Enzymes

**FIGURE 3:**
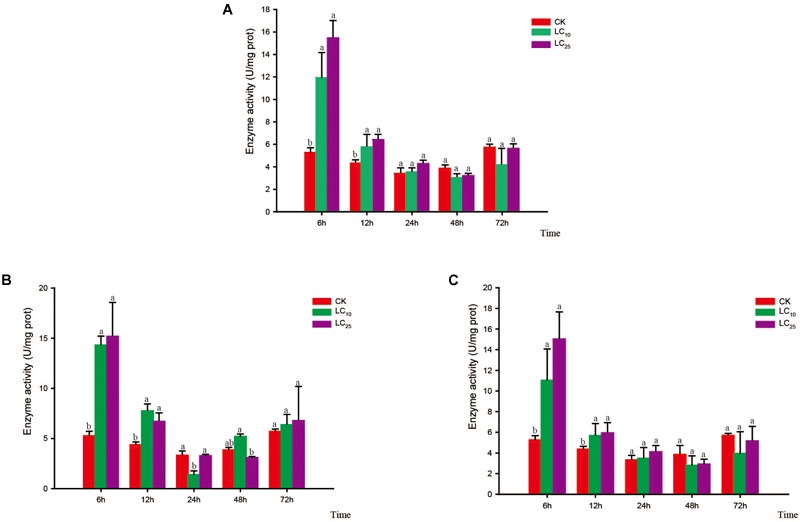
Effects of sublethal concentrations of insecticides on the carboxylesterase (CarE) activity of *Sogatella furcifera*. Enzyme activities are shown as the mean ± SE. Different letters indicate significant differences among treatments at the same time. **(A)** Thiamethoxam; **(B)** buprofezin; and **(C)** abamectin.

**FIGURE 4:**
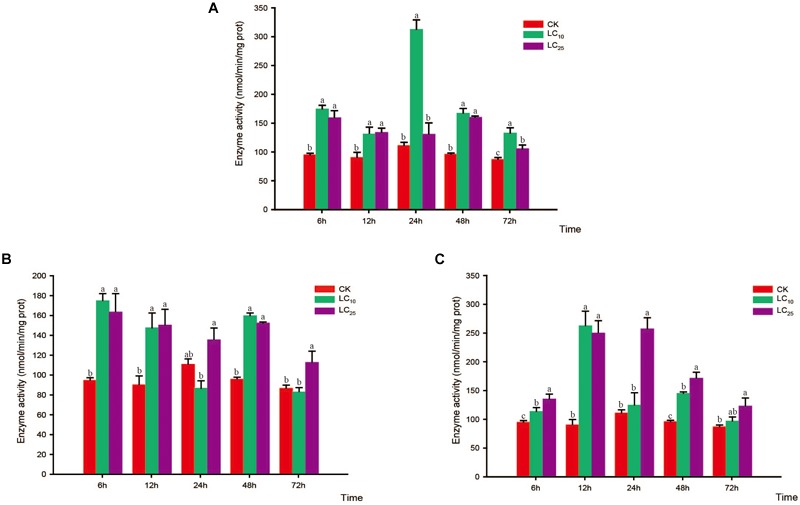
Effects of sublethal concentrations of insecticides on the glutathione *S*-transferase (GST) activity of *Sogatella furcifera*. Enzyme activities are shown as the mean ± SE. Different letters indicate significant differences among treatments at the same time. **(A)** Thiamethoxam; **(B)** buprofezin; and **(C)** abamectin.

**FIGURE 5:**
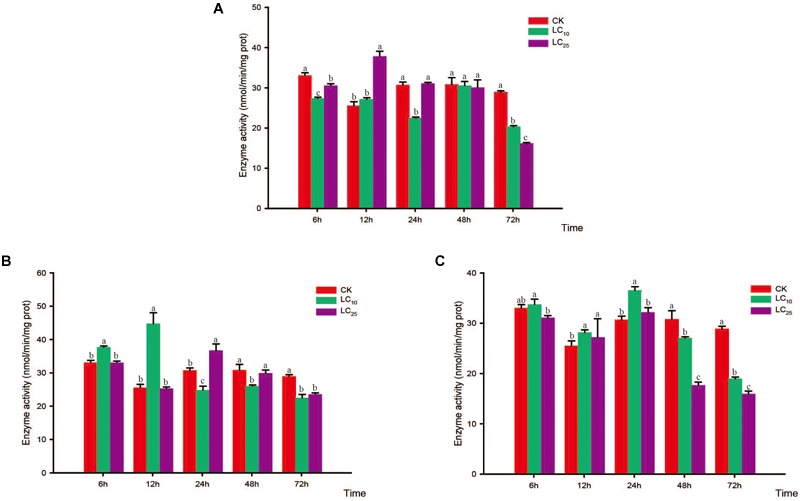
Effects of sublethal concentrations of insecticides on the mixed-function oxidase (MFO) activity of *Sogatella furcifera*. Enzyme activities are shown as the mean ± SE. Different letters indicate significant differences among treatments at the same time. **(A)** Thiamethoxam; **(B)** buprofezin; and **(C)** abamectin.

Changes in the activity of the detoxifying enzymes in *S. furcifera* were examined after treatment with sublethal concentrations of the test insecticides for 6, 12, 24, 48, and 72 h (Figures 3–5). Compared with control levels, the activity of CarE was significantly increased after 6 and 12 h of treatment with thiamethoxam, buprofezin, and abamectin at the LC_10_ and LC_25_ levels, showing the same trend for all three insecticides and with the activity being highest at 6 h (Figure 3). It is worth noting that after treatment with the three insecticides at LC_10_ and LC_25_, there was an initial increase in the overall activity of CarE with time, followed by a decrease, and then subsequently a further increase.

Glutathione *S*-transferase activity increased gradually and then decreased after treatment with thiamethoxam at LC_10_, peaking at 24 h (2.8-fold higher than that of the control). However, in response to treatment with thiamethoxam at LC_25_, there was no significant difference between the GST treatment and control groups after 24 h, and activity of the enzyme returned to normal levels at 72 h (Figure 4A). After treatment with buprofezin at LC_10_ and LC_25_, GST activity showed an overall increasing trend, being highest at 6 h after the LC_25_ treatment, and subsequently decreasing with the prolongation of treatment time, albeit at levels significantly higher than that of the control. In contrast, we observed a significant reduction in GST activity in response to treatment with buprofezin at LC_10_ for 24 h (Bonferroni-corrected *P =* 0.04) compared with that of the control, although again the levels had returned to normal at 72 h (Figure 4B). In response to treatment with abamectin at LC_10_ and LC_25_, the activity of GST increased significantly at 6 h, reached a maximum at 12 h, and then gradually decreased. Compared with control levels, the activity of this enzyme was significantly higher in response to the LC_25_ treatment. However, similar to the response to buprofezin treatment at LC_10_, GST activity following abamectin treatment at LC_10_ was not significantly different from that of the control at 24 and 72 h (Figure 4C).

Compared with the control, the activity of MFO showed a decreasing trend in response to treatment with thiamethoxam at LC_10_, with the difference being significant at 6, 24, and 72 h. In contrast, in response to treatment with thiamethoxam at LC_25_, although the activity of MFO had decreased at 6 and 72 h, we observed a significant increase at 12 h (Bonferroni-corrected *P =* 0.001) relative to the control level (Figure 5A). In response to buprofezin exposure at LC_10_, MFO activity was significantly increased at 6 and 12 h compared with that of the control, and reached a peak at 12 h (1.8-fold higher than that of the control). However, at 24, 48, and 72 h, the activity of MFO was significantly reduced. In addition, after treatment with buprofezin at LC_25_, we detected no significant difference between treatment and control MFO activities at 6 and 12 h, whereas there was a significant increase in activity in response to treatment at 24 h, which thereafter gradually decreased (Figure 5B). In response to treatment with abamectin at LC_10_ and LC_25_, MFO activity showed a decreasing trend compared with the control levels, with the difference being significant at 48 and 72 h. However, in response to abamectin treatment at LC_10_, MFO activity was significantly higher than that of the control after 12 and 24 h (Figure 5C).

### Activity of Protective Enzymes

**FIGURE 6:**
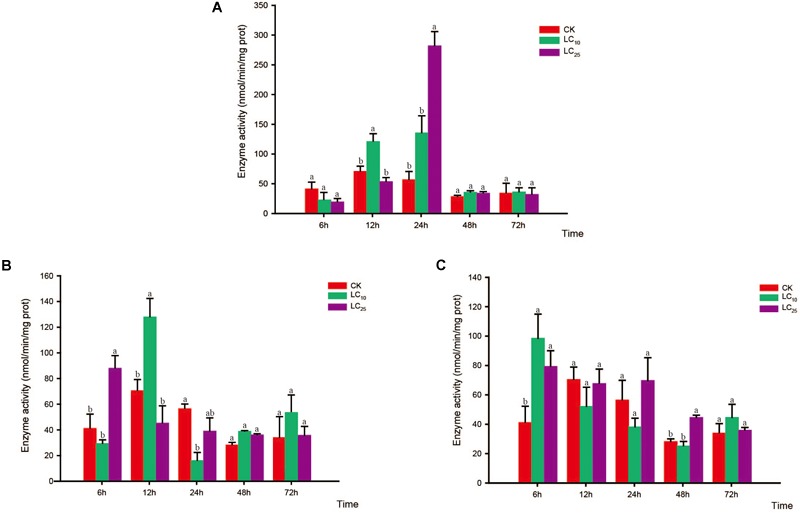
Effects of sublethal concentrations of insecticides on the catalase (CAT) activity of *Sogatella furcifera*. Enzyme activities are shown as the mean ± SE. Different letters indicate significant differences among treatments at the same time. **(A)** Thiamethoxam; **(B)** buprofezin; and **(C)** abamectin.

**FIGURE 7:**
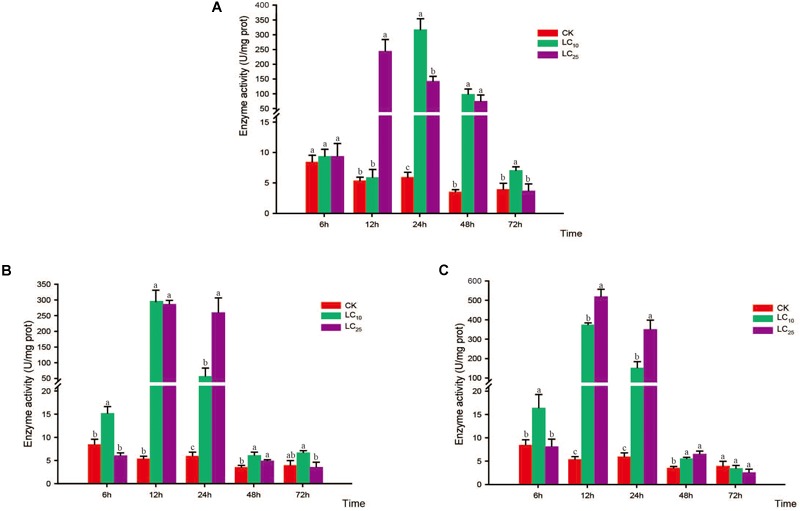
Effects of sublethal concentrations of insecticides on the peroxidase (POD) activity of *Sogatella furcifera*. Enzyme activities are shown as the mean ± SE. Different letters indicate significant differences among treatments at the same time. **(A)** Thiamethoxam; **(B)** buprofezin; and **(C)** abamectin.

**FIGURE 8:**
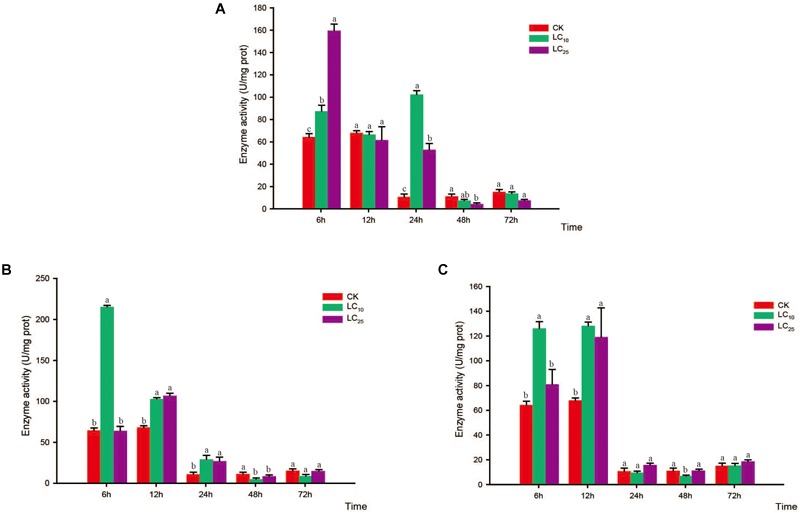
Effects of sublethal concentrations of insecticides on the superoxide dismutase (SOD) activity of *Sogatella furcifera*. Enzyme activities are shown as the mean ± SE. Different letters indicate significant differences among treatments at the same time. **(A)** Thiamethoxam; **(B)** buprofezin; and **(C)** abamectin.

The activities of CAT, POD, and SOD were measured at 6, 12, 24, 48, and 72 h after exposure to sublethal concentrations of the test insecticides (Figures 6–8). Although at 6 h after treatment with thiamethoxam at LC_10_ and LC_25_, we observed an inhibition of CAT activity, at 12 and 24 h the activity had increased significantly, respectively, but thereafter returned to normal levels (Figure 6A). Following treatment with buprofezin at LC_10_ and LC_25_, CAT activity had increased significantly by 1.8- and 2.1-fold at 12 and 6 h, respectively, compared with the control, and in the LC_25_ treatment group thereafter gradually returned to a normal level (Figure 6B). Similarly, after treatment with abamectin at LC_10_ and LC_25_, CAT activity was 2.4- and 1.9-fold higher, respectively, than that of the control at 6 h, and in the LC_25_ treatment group subsequently underwent a gradual return to normal levels. However, after 48 h of LC_25_ treatment, the activity of this enzyme had increased significantly to a level 1.6-fold higher than that of the control (Figure 6C). Interestingly, in response to treatment with both buprofezin and abamectin at LC_10_, CAT activity initially increased, then decreased, and subsequently increased again with a prolongation of exposure time.

In response to treatment with thiamethoxam at both LC_10_ and LC_25_, POD activity showed a tendency to initial increase and subsequently return to a normal level (Figure 7A). In the case of the LC_10_ treatment, POD activity peaked at 24 h (53.7-fold higher than that of the control) and then gradually decreased, albeit at levels still significantly higher than those of the control. In the LC_25_ treatment group, POD activity peaked at 12 h (45.9-fold higher than that of the control) and then decreased gradually until reaching the normal level at 72 h (Figure 7A). After treatment with buprofezin at LC_10_, POD activity began to increase significantly at 6 h (Bonferroni-corrected *P =* 0.004), peaked at 12 h (55.7-fold higher than that of the control), and then decreased gradually to a normal level after 72 h. Similarly, after treatment with buprofezin at LC_25_, POD activity showed a significant increase at 12 h (53.9-fold higher than that of the control) (Wilcoxon signed-rank test *P* < 0.05) and then gradually decreased to a normal level after 72 h (Figure 7B). The responses of POD activity following exposure to abamectin at LC_10_ and LC_25_ showed similar patterns to those following buprofezin treatment at LC_10_ and LC_25_, whereby activity peaked at 12 h (70.3- and 97.7-fold higher than that of the control, respectively) and returned to a normal level after 72 h (Figure 7C).

Compared with the control level, the SOD activity levels following thiamethoxam treatment at LC_10_ and LC_25_ were significantly increased at 6 h (1.4- and 2.5-fold higher than that of the control, respectively) and returned to normal levels at 12 h. Subsequently, however, the SOD activity showed a secondary significant increase at 24 h, before eventually returning to a normal level thereafter (Figure 8A). In response to treatment with buprofezin at LC_10_, SOD activity increased significantly at 6 h (3.4-fold higher than that of the control), and then underwent a gradual decrease (Figure 8B), whereas following treatment at LC_25_, the activity of this enzyme increased significantly at 12 h to a level 1.6-fold higher than that of the control. After 48 h of exposure to buprofezin at LC_10_ and LC_25_, SOD activity had decreased by 59.9 and 26.5%, respectively, compared with the control level, but had returned to a normal level at 72 h (Figure 8B). The responses of SOD activity to treatment with abamectin at LC_10_ and LC_25_ showed trends similar to those following buprofezin treatment at LC_10_ and LC_25_; however, after 24 h of abamectin treatment at both sublethal concentrations, SOD activity showed a tendency to return to a normal level (Figure 8C).

## Discussion

Previous studies on insects have shown that the protective enzymes SOD, POD, and CAT are related to resistance and the response to insecticide-induced stress. In this regard, it has been reported that sublethal concentrations (LC_10_ and LC_25_) of abamectin can promote upregulation of the SOD, POD, and CAT activities in *Diadegma semiclausum* adults, with activity increasing with increasing insecticide concentration (Jia et al., 2016). In third-instar *H. axyridis* nymphs exposed to LC_10_ abamectin, the highest levels of SOD, POD, and CAT activity were recorded at 24, 12, and 24 h, respectively, and were significantly higher than those in the control group (Yang et al., 2015). In the present study, the overall levels of SOD, POD, and CAT activity in abamectin-treated (LC_10_ and LC_25_) *S. furcifer* tended to undergo an initial increase and thereafter gradually return to normal levels, reaching their highest levels at 12, 12, and 6 h, respectively. Interestingly, at 12 and 24 h, POD and CAT activities showed an increase in response to increasing abamectin concentration, which is consistent with the observations on *D. semiclausum* previously reported by Jia et al. (2016). In contrast, the levels of SOD, POD, and CAT activity in third-instar *H. axyridis* nymphs were shown to decrease with an increase in abamectin concentration (Yang et al., 2015). In the present study, we found that exposure to thiamethoxam initially tended to promote upregulation of the overall activities of POD and SOD and then inhibit them with an increase in the insecticide concentration from LC_10_ to LC_25_, which contrasts with the observations for buprofezin (LC_10_ and LC_25_), which initially inhibited and then upregulated POD and SOD activities with increasing sublethal concentration. For CAT, buprofezin initially upregulated and then inhibited enzyme activity with increase in concentration, whereas thiamethoxam tended to initially inhibit and then upregulate CAT activity with increase in concentration. Similar observations have previously be made in *Aphidius gifuensis*, in which the levels of SOD, POD, and CAT activity tended to decrease with an increase in imidacloprid concentration (LC_10_, LC_20_, LC_30_, and LC_50_) (Zhu et al., 2015). Such studies indicate that, in insects, SOD, POD, and CAT activities are related to insect resistance and the response to insecticide-induced stress, although the effects of these enzymes may be species, concentration, and time dependent.

The detoxifying enzymes CarE, GST, and MFO are also important components of insect resistance mechanisms, an increase in the activities of which is necessary during insecticide metabolism (Qi et al., 2016). Previously, it has been found that the levels of GST and MFO activity in two color morphs of the pea aphid *Acyrthosiphon pisum* increased in response to increasing sublethal concentrations of abamectin (LC_5_, LC_10_, and LC_20_) following exposure for over 24 h (Wang and Liu, 2014). Similarly, the activities of CarE, GST, and MFO in *Tetranychus urticae* were significantly upregulated at 12 h following exposure to abamectin (LC_10_ and LC_25_) (Ru et al., 2017). In the present study, avermectin (LC_10_ and LC_25_) resulted in a similar significant induction of CarE, GST, and MFO activities in *S. furcifera*, at 6, 12, and 24 h, respectively. These findings indicate that insects can adapt to the stress induced by avermectin by activating their detoxifying enzymes. In addition, after treatment with thiamethoxam and buprofezin (LC_10_ and LC_25_), CarE activity showed an overall trend of initial upregulation and subsequent inhibition, with the activity being highest at 6 h. Thiamethoxam and buprofezin also significantly induced GST activity in *S. furcifera*, whereas these insecticides were found to have a generally inhibitory effect on the activity of MFO. Previously, it was found that GST and P450 activities in *Aphis craccivora* were significantly induced after treatment with cycloxaprid and imidacloprid (LC_50_) for 48 h, whereas in contrast, the activity of the CarE activity was inhibited, although the observed difference was not significant (Wu et al., 2016). In addition, after treating *Cydia pomonella* with imidacloprid (LC_20_), Shang et al. (2017) observed a significant induction of CarE and GST activity, whereas MFO activity was significantly inhibited. These findings suggest that MFO may not play a major role in the insect response to stress induced by neonicotinoid insecticides, and that the primary detoxifying enzymes are CarE and GST. The aforementioned findings indicate that detoxifying enzymes enable insects to respond to low levels of insecticide-induced stress; however, similar to protective enzymes, CarE, GST, and MFO are induced at different times in different insects. Moreover, the main enzymes involved in detoxification appear to be species dependent.

The ABC transporters are important participants in the third stage of detoxification and have been widely reported to be involved in insecticide resistance (Qi et al., 2016). In this regard, it has previously been found that the expression levels of an *ABCG* gene and an *ABCC* gene were upregulated in *S. furcifera* treated with a high concentration (LC_85_) of cycloxaprid, whereas the expression levels of two *ABCG* genes were upregulated at a low concentration (LC_15_) of this insecticide (Yang et al., 2016). Transcriptome sequencing has revealed that the *ABCB, ABCC*, and *ABCG* subfamily genes are expressed at high levels in a pyrethroid-resistant strain of *Aedes aegypti* (Bariami et al., 2012). Similarly, results of microarray experiments have shown that genes of the *ABCG* and *ABCH* subfamilies are expressed at high levels in resistant strains of *Myzus persicae* (Silva et al., 2012b), and that the expression levels of *ABCG* subfamily genes are increased in DDT-resistant strains of *Anopheles arabiensis* (Jones et al., 2012). Given that *ABCG* subfamily genes play a role in insecticide resistance in many insects (You et al., 2013; Yang et al., 2016; Sun et al., 2017; Xiao et al., 2018), we decided to study the expression of 14 *ABCG* subfamily genes in *S. furcifera* in response to thiamethoxam, buprofezin, and abamectin. We accordingly found that 13 of these 14 *sfABCG* genes were significantly upregulated after treatment with at least one sublethal concentration of insecticide. On exposure to these insecticides at the LC_10_ level, 13 *sfABCG* genes were significantly upregulated by thiamethoxam, 12 were significantly upregulated by thiamethoxam and buprofezin, and nine were upregulated by all three insecticides. Furthermore, 13 and seven *sfABCG* genes were significantly upregulated after treatment with LC_25_ concentrations of thiamethoxam and abamectin, respectively. These findings provide further evidence that ABC transporters probably participate in the transport of various substrates related to the resistance to different types of insecticides. Moreover, it is conceivable that, in addition to enhancing the metabolism of *S. furcifera*, these highly expressed *sfABCG* genes are associated with cross-resistance in this insect. However, these inferences need to be verified with functional experiments.

The sublethal effects of insecticides on insects are multifaceted, including their effects on insect behavior, reproduction, development, and insecticide resistance. In addition, insect adaptation to insecticide stress is a complex metabolic detoxification process involving the activity of multiple enzymes. The results of our study show that *S. furcifera* can eliminate insecticides in the body by activating detoxifying enzymes and ABC transporters, and also activate the protective enzyme system to prevent injury to the body. Taken together, our research results lay the foundations for gaining a deeper understanding of the mechanisms contributing to the adaptation of *S. furcifera* to different types of insecticides, which is of considerable significance with regards to the development of effective pest management strategies.

## Data Availability Statement

The gene sequences obtained have been submitted to the NCBI database (Accession Nos. MH481837–MH481850). Other datasets for this study are included in the manuscript and the Supplementary Files.

## Author Contributions

HY conceived and designed the experiments. ZW and G-YL measured the detoxifying and protective enzyme activities. CZ examined the *ABCG* gene expression levels and prepared the manuscript. CZ, HY, ZW, D-CJ, and G-YL finalized the manuscript. All authors read and approved the final manuscript.

## Conflict of Interest Statement

The authors declare that the research was conducted in the absence of any commercial or financial relationships that could be construed as a potential conflict of interest.

## Footnotes

1 http://www.ncbi.nlm.nih.gov/

2 http://www.ncbi.nlm.nih.gov/orffinder/

3 http://pfam.xfam.org/

4 https://www.ncbi.nlm.nih.gov/cdd
