# Supplementary material for: Protective and Detoxifying Enzyme Activity and ABCG Subfamily Gene Expression in Sogatella furcifera Under Insecticide Stress
Source: Front Physiol. 2019 Jan 8;9:1890. doi: 10.3389/fphys.2018.01890 (PMC6331518; doi:10.3389/fphys.2018.01890)
Supplement: Supplementary file 1 [file Data_Sheet_1.docx]

**Supplementary Material**

**Effects of Insecticides on** [**Protective**](http://cn.bing.com/dict/search?q=Protective&FORM=BDVSP6&mkt=zh-cn)**and Detoxifying Enzyme Activity, and Expression of ABCG subfamily, *Sogatella furcifera***

**Cao Zhou^1^, Hong Yang^1, 2*^, Zhao Wang^1, 3^, Gui-yun Long^1^, and Dao-chao Jin^1^**

^1^Institute of Entomology, Guizhou University; Provincial Key Laboratory for Agricultural Pest Management of Mountainous Regions, Guiyang 550025, People’s Republic of China

^2^College of Tobacco Science of Guizhou University, Guiyang, 550025, People’s Republic of China

^3^College of Environment and Life Sciences, Kaili University, Kaili, 556011, People’s Republic of China

*** Correspondence:**

Pro. Hong Yang

maximus@gmail.com

E-mail address: [axyridis@163.com](mailto:axyridis@163.com)

Supplementary Table S1. LC10 and LC25 values of thiamethoxam, abamectin, and buprofezin against *Sogatella furcifera*.

| Insecticides | LC_10_ values (mg/L) | LC_25_ values (mg/L) |
| --- | --- | --- |
| Thiamethoxam | 0.0332 | 0.1428 |
| Abamectin | 1.0446 | 2.1551 |
| Buprofezin | 0.1186 | 0.3478 |

Supplementary Table S2. The primers of ABC transporter G subfamily gene and 18S gene for RT-qPCR.

| Gene | Forward (5′–3′ ) | Reverse (5′–3′ ) |
| --- | --- | --- |
| *SfABCG1* | ATCTTCCTCGATGAGCCAACCA | CGGAGTGTGGATGGAGCAGAT |
| *SfABCG2* | TTGCCATGTCCAGGAGGAGTAG | GGTGACGGCTGCTAGTTGATG |
| *SfABCG3* | TCAAGGCGGACGCACAATAGT | AGGCTGTGAGTGGCAAGGAAG |
| *SfABCG4* | TTGAGCTTCTCACTGAGCCTTC | GCACACCACCATCACTCCTT |
| *SfABCG5* | CACGCCAGTCAGCAGCATT | CGGCATCTGTTGTCTGAGAGG |
| *SfABCG6* | GTTACCGTGTCCGACCTACCA | CCGTTCTCTGTTCCATCCACAA |
| *SfABCG7* | GGCTCGGTGAAGGTGAACATC | CCTCGGACACGGAGTAGTTGA |
| *SfABCG8* | TCCGCCGATCCTGTTCCTTGA | GCAAACGATTGTGTGCCCTTCA |
| *SfABCG9* | CGAGATGAGGCAAGGTTCAGGA | TTGAGTAGGGCGGCAAAGTTCA |
| *SfABCG10* | TCAGGTGGCAAGACGCAGTGTA | TGGCTCGTCCAGGAGGAGTAGA |
| *SfABCG11* | GACAGCTCCAGCACCGTTCA | AACACATCGTCCGCCGTTGAT |
| *SfABCG12* | CAGCCATCTACAGAGCCTAGCA | CTCGCCGTCAGACAGCAAGT |
| *SfABCG13* | GCTGGAAGAGGCGGATATGCT | TTGGGCGTGGGACAGTGAGT |
| *SfABCG14* | AGGAGTATGGTGAGCCGCAATG | AGGGCGATCAGTTCTCCAGGAT |
| *SfRPL9* | GGGCGAGAAGTACATCCGTAGG | GCGGCTGATCGTGAGACATCTT |
