# Supplementary material for: Protective and Detoxifying Enzyme Activity and ABCG Subfamily Gene Expression in Sogatella furcifera Under Insecticide Stress
Source: Front Physiol. 2019 Jan 8;9:1890. doi: 10.3389/fphys.2018.01890 (PMC6331518; doi:10.3389/fphys.2018.01890)
Supplement: Supplementary file 2 [file Data_Sheet_2.docx]

**Supplementary Material**

**Effects of Insecticides on** [**Protective**](http://cn.bing.com/dict/search?q=Protective&FORM=BDVSP6&mkt=zh-cn)**and Detoxifying Enzyme Activity, and Expression of ABCG subfamily, *Sogatella furcifera***

**Cao Zhou^1^, Hong Yang^1, 2*^, Zhao Wang^1, 3^, Gui-yun Long^1^, and Dao-chao Jin^1^**

^1^Institute of Entomology, Guizhou University; Provincial Key Laboratory for Agricultural Pest Management of Mountainous Regions, Guiyang 550025, People’s Republic of China

^2^College of Tobacco Science of Guizhou University, Guiyang, 550025, People’s Republic of China

^3^College of Environment and Life Sciences, Kaili University, Kaili, 556011, People’s Republic of China

*** Correspondence:**

Pro. Hong Yang

maximus@gmail.com

E-mail address: [axyridis@163.com](mailto:axyridis@163.com)


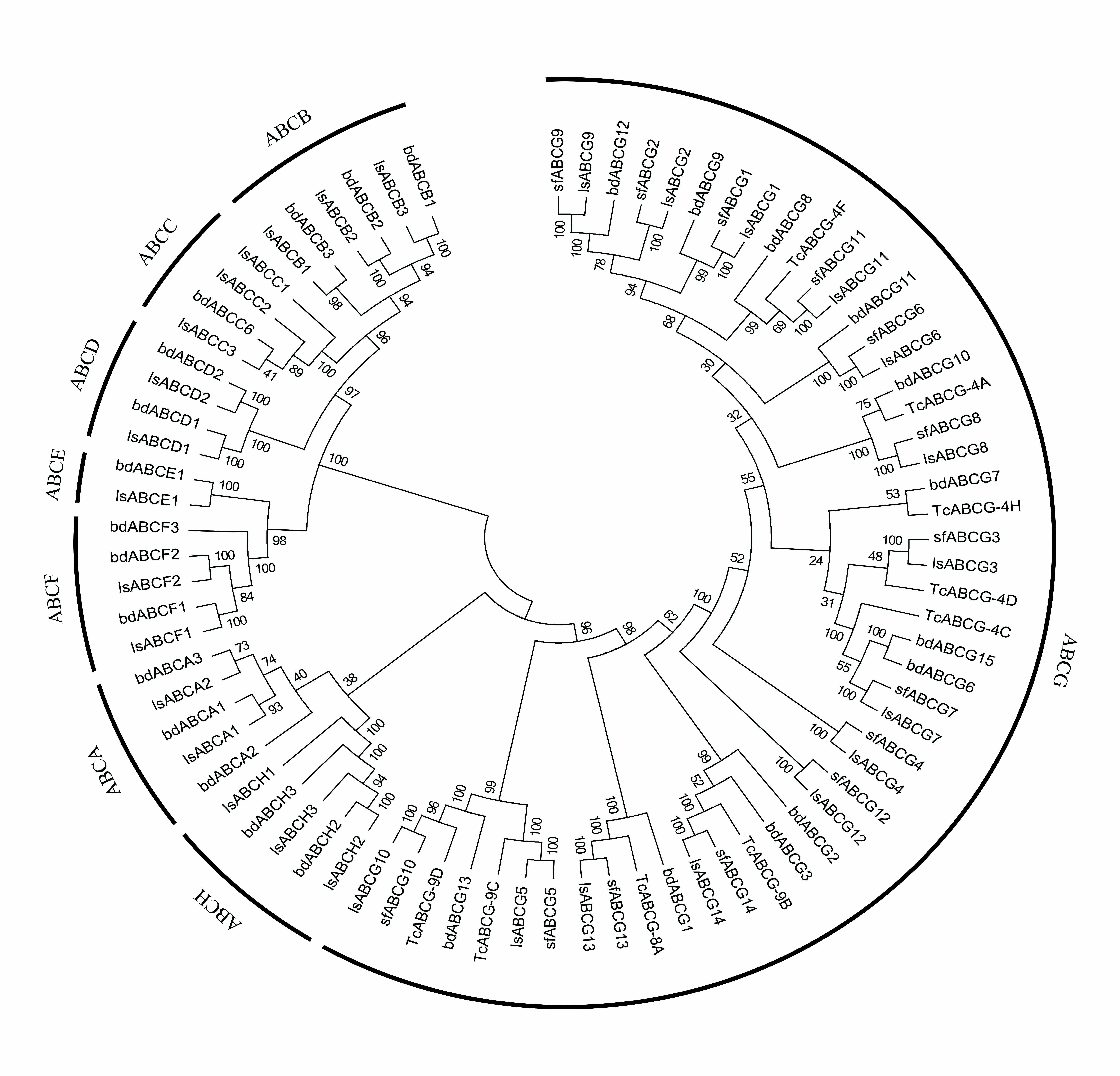


**Supplementary Figure S1**. Phylogenetic analysis of ABC transporter G subfamily genes in *Sogatella furcifera*, *Laodelphax striatellus,* *Tribolium castaneum*, and *Bactrocera dorsalis*. ls, *L. striatellus*; sf, *S. furcifera*; Tc, Tribolium castaneum; and bd, Bactrocera dorsalis.


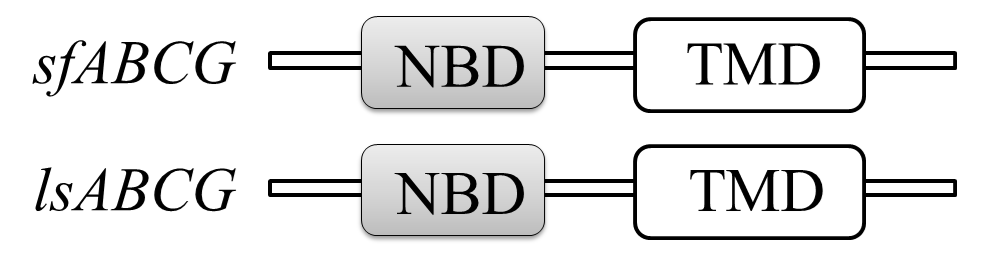


**Supplementary Figure S2**. Characteristic domains of ABC transporter G subfamily genes in *Sogatella furcifera*, *Laodelphax striatellus*. Abbreviations : NBD, nucleotide binding domain; TMD, trans-membrane domain; ls, *L. striatellus*; sf, *S. furcifera*.
