# Supplementary material for: Protective and Detoxifying Enzyme Activity and ABCG Subfamily Gene Expression in Sogatella furcifera Under Insecticide Stress
Source: Front Physiol. 2019 Jan 8;9:1890. doi: 10.3389/fphys.2018.01890 (PMC6331518; doi:10.3389/fphys.2018.01890)
Supplement: Supplementary file 3 [file Data_Sheet_3.docx]

**Supplementary Material**

**Effects of Insecticides on** [**Protective**](http://cn.bing.com/dict/search?q=Protective&FORM=BDVSP6&mkt=zh-cn)**and Detoxifying Enzyme Activity, and Expression of ABCG subfamily, *Sogatella furcifera***

**Cao Zhou^1^, Hong Yang^1, 2*^, Zhao Wang^1, 3^, Gui-yun Long^1^, and Dao-chao Jin^1^**

^1^Institute of Entomology, Guizhou University; Provincial Key Laboratory for Agricultural Pest Management of Mountainous Regions, Guiyang 550025, People’s Republic of China

^2^College of Tobacco Science of Guizhou University, Guiyang, 550025, People’s Republic of China

^3^College of Environment and Life Sciences, Kaili University, Kaili, 556011, People’s Republic of China

*** Correspondence:**

Pro. Hong Yang

maximus@gmail.com

E-mail address: [axyridis@163.com](mailto:axyridis@163.com)

>*sfABCG1*

ATGGATAATCCTCCGACTGTGGCCAATATAAACCACCTCATTCAGAGGCCACCGGTTGATATAGAGTTCACAGACCTAACATACACAGTTCCTCATGGAAGAAGTGGATCCAAGATTATCCTGAGAAGTGTGAGTGGCTTATTCAAATCAGGACAGTTGACAGCGATCCTGGGACCGTCTGGAGCTGGGAAGAGCACGCTTCTAAATGTCCTCGCTGGATATAAATGCGCGGACTCAACTGGTTCCATTCTGGTCAACGGACGTCCAAGAGTTCTGCAACAGTTTCGAAAATTATCCAGATACATAATGCAGGAAGATATGCTGCAGCCTCGGCTCACTGTGCAGGAATCCATGCTGTTCGCTGTCGATTTGAAACTTGGAACCACCATTTCACAAGAAGAAAAGCTTGATACTATTGATGAGATTTTGAACATGTTGAGGCTTTCGAAGACGAAGAACACTCTGTCCGGCCATTTGTCAGGTGGTGAGAAGAAAAGGCTGTCCATTGCACTGGAACTAGTGAACAATCCTCCTGTCATCTTCCTCGATGAGCCAACCACGGGCTTGGATGATTTGGCCAGTTCACAATGCATATCCCTGTTGAAAGCGCTGGCGAGAGGGGGTCGCACAGTGATCTGCTCCATCCACACTCCGAGCGCGCGACTCTTCTCGCTGTTCGATCATGTGTACGTTGTGTCGGAAGGCCAGTGTGTGTTCCAAGGCCATGGACACGATATTGTCGATTTTTTGGCCTCATTCGGTTTGAACTGTCCCAAGCACTATAATCCTATTGATTTCATGATTGAAGTATCAAGTGGAGAGTATGGTGATTATCTCGAGAGAATGACGAATGCCGTTGAGAATGGGAGATGCTACAGGTGGAGTCAAAACAAAGTCACTGATGTCCGATATCAATCAACAAATGAAGAGGAAGACAATCTAGTCAGCTCAGATCTCCATCACATGTATCATTTTGAAAGTTCAGCGTGGTTGCAGTTCAGAATATTAATTAATAGGATGTCTCTACAAGGTAGAAGAGATATGGGATATATTATATTGAAATTAGCAATGCATATTTTCATAGGACTTATAATTGGAGGGATGTTCTTCCAAATTGGAAACGATGGATCAAAGACTATTTTCAATTTCGGTTTCTGTTTTGTAACTATTATCATTTTCTTGTACATACCGATGATGCCAGCGCTTCTATGGTTTCCACAAGAAGTGCAGCTATTGAAGAGAGAATTCTTCAATCGATGGTATGATCTTAATCCATACTTCTTTGCCATGACATTCTGCCAGTTACCGCTGCAAATTGTTTTTGGAATCGGTTACACGTTACTAACTTACTTCATGACTGATCAACCAATGGAGTATGAGAGAGTTCTGAAATTCATCCTGGTGTGCCTGATGATCTCTATTGTGTCAGAAGCCATGGGCTTGGCTATATCTGCTCGACTGAATATTGTGAACGGAATATTCGTAGGGCCGGCTGTTTCAGTTCCCCTGATGTTGTTGGCTGTTTACGGCCTGGGCACAGGCAGCAAATACATCCCGAAACACATTCGATTCGCCATGTACTTCAGCTATTTGCGCTACGGCTTGGAAGGACTGATATCCTCCATATACGGCGGCGGACGTAGGAAAATGGTCTGCCCCGATTCGGAAATCTACTGTCAGCTGAGAGAACCGAAAGCGCTACTGAAGGAAGTTGGTATGGAAGATGTTAATTATTGGATGGATATAGCTGCCTTAGCTGTGTCATTTTTGGTTTTCAAGATTATCTGTTATGTACTGTTGAGAAGACGGTTAAAGTCGACCCAATCTTTTGGAGCCCTTGGTTTTATTGGCAGATTCATCAAGACCCATTTCAACCTAGCTGGAAATATAGGAAGGTAA

>*sfABCG2*

ATGTCAGCTTCAACTTCGTCAGCACTGGAGAGTATTGTTGAAGTTTTGCCATGTCCAGGAGGAGTAGGAGACGCCGGAGTCGAGCCGTTACCGGGCTGCAGCGGCGAAGGGAACGCCAAGGGCGCACCATCAACTAGCAGCCGTCACCCGCCCGTGCCCGTCACACGGATTCCAACGCGGCTCGGTCTCACCACGTTGACCAGAATGGCGAAACGGCCCGCAGTCGACATCGAGTTCCAGGATCTCTCCTACACGGCTGGAAGTCGAAAGATTTTGAAATCGATATCGGGATGCTTCAAATCGGGAGAGATGACCGCCATCATGGGTCCTTCAGGAGCTGGAAAGAGTACCTTGATGAATATCCTTGTCGGATATGTGACAAATGGAGTATCGGGAAGTATAATGACAAACGGATTCCCACGACAAATCAAGCTGTTCAACAAGCTGTCATCCTACATAATGCAAGAAGACCTGTTGCAGCCCAACCTCACTGTCAGGGAGTCCATGATGATTGCTGCCAGACTAAAATTGGGAAACGAATTATCGGACAGGGATAAGAATGCTGCTGTGAGAGAAATTCTAGTGACCTTGGGTTTGACAAAGTGCGCGGATACGTTTACAGACCGTCTGTCGGGCGGTCAGCGCAAGAGGGTGTCTGTGGGACTTGAACTTGTCAACAATCCACCGGTCATATTTCTGGACGAACCGACCACAGGTCTAGACATAGTGGCCATCAATAACTGTATAGAATTGTTGAAAGACCTATCAAGTCAGGGCAGGACCATTGTGTGTACCATCCACCAGCCCACTGCATCCATGTTCAACATGTTCGATAATGTATACATGCTAGCCAAAGGCCAATGTATCTATCACGGCACTTCGCATCAACTGGTGCCTTTCCTCAGCAGTTGCAATCTTGACTGTCCCCCCACCTATAATCCAACCGATTTCGTTTTTGAAGTTTTGGAAGCAAATCCAGAATTAATTAAAGTGATGAACTCAGAAATACAGAATGGAAGAGTCATCTGGTTGGATCCAACAGATAACCCTGAGCCAAAGTCAAAACTATGTAGGAAAGATACCCTAGCTGTGATGCCCAATGTTATTGGATCGGATAGTGTGATACACTTCCCAACATCATTTTTCGAACAAGTAACAATCTTGCTGAAAAGAATGATGAAACAAAAATGGAGAAATTCGACTGCCATGAGGTTGCAAATGATCCATCATTTGTTTTCTGGACTGATTGTTGGTTCAATATTCTATGGAATTGGAAATAATGCCAGCAAGCCGTTCGAAAATTTCAAATTTGTTCTTTGTGTAGCTGTTTTCTTCATGTATACGCATGTTATAACGCATATCTTGACTTTGCCAAATGAAATAAAAATAATGAAAAGAGAGTATTTCAACCGATGGTATGGACTCAAAGCCTATTTCACGGCACTCACCCTACATACAGTTCCTACTACTATAATTTTGGGCATGTTATTCAACACAATTGTCTACATAATGGCGGATGAACCATTGGAGCTTCCCAGGTTCATATGGTTCAGTTCGTTTACAATAATGGTGGCTCTAGTCTCAGAGGGACTGGGTGTACTTATCGGCTGTAACTTCAATTGCACGAACGGAGCCGTAGTCGGTCCCTCAGTGATGGCGCCCATACTGATGATCGCCATCCACGGCATGGGTTACGGGCTGCACATCAAGCCCTTCATGCAGAGCTTAATGAAGCTGAGCTTCATCCGCGTGGCGGTGGTGGGCATAGTGACCAGCCTTTACGAGAACGGTCGTGGTCCCATGGAGTGCAAAACGCAGACGCATCCCTACTGCCACTACCGCGACCCCTACATGCTGGTCAGAGACCTGGGCATGACCAATCAGAGCACAGTGCATCAGATTTTAGGTCTGATCGGGTTTCTGCTGCTGTTCAGGACGGCCGCGTTTTTGACGCTGCGCTACACACTGATGACGGACATCAGGAGTCAGGTGTTTGCATACACTAAGAAAATATTCAAGCGCAACAAGGAGAAGCGCCTGTTGTTGTCCGATGACAAGTAG

>*sfABCG3*

ATGTCCGGCATATTCAAGCTATTCAATCTCAAGACTGGGAGCGTTCAAAAGGAGTCATCGCATAACATCTACACGGTGGACCTAACGCAGGATGGCACAGACCAACAATCGGCTTTCTCGCTCAAGCACCTCCCGAAACGACAGCCAGTCGATATCAAATTTATCGATTTGAATTACTCAGTACCTGACAATTCGCGCTCCGGTTCGAAAAAGATTCTAAATGATTTGCAAGGAGAATTCAGGTCTGGAGAATTGGTGGGAATACTGGGACCGTCCGGCGCTGGGAAAAGTACACTGCTCAACGCCTTAGTGGGGTTTGGAACCAAAGGCAGGTCGGGAACAATACTGGTGAACAATCAAGTGATGGACGCCGAATCGTTTCGTAAAGTGAGCTGCTACATAATGCAAAAAGGCGAGCTACTGCCCTACCTGACTGTCGGCGAGGCTATGATGGTATCCGCCAACCTCAAACTGGGGACATCGGTCAGCAAATCCGAGAAAAAAGTTATTATAGACGAGATTCTAACAGCAATTGGTCTGGATAAACATATCGACACGTACTGTAAAAATCTATCTGGCGGACAGAAGAAACGACTCCTGGTGGCTGTGGAACTAGTTTATAATCCACCTCTCATGTTCCTTGATGAGCCTACAAGCGGCCTAGACAGTTCATCTAGCGTACAGTGTGTGTCGCTGCTGAAATCGCTAGCTCAAGGCGGACGCACAATAGTGTGCACCATTCACCAGCCAAACGCGCGCACATTTGAATTGTTCGATCAGCTGTTTGTACTGGCACCCGGACACTGCATCTACCAGGGCCCTGTGCACTCGCTTGTCCCCTTCCTTGCCACTCACAGCCTCATCTGTCCCAGCTACCACAATCCGGCCGATTTCGTAATTGAAGTTGCAATGGGACAACATGGACCAATCCAGGCTCTCACAAAGGAGGTTAATAGAAACATAAAGGAGAAATTCAAGGAACAAGAGAACAGAATATGTGATAAGATCAATGAAATTGACCAACCAAACAATAGAAATTCCATATCAGGCGAAAAATTCAGGATATTATCCAGTGAGCTTGAGCTATCTCCAAGAGACGAGAACTCTTTCAATAGTCAAAAAAACGAAGGAGAATTCGATGTTTGTGAGAGACCCTCAAGCTTATTCCAGTTCTATGTTTTGTTGAGAAGAACACTCACGTCAACATTAAGAGATATTCATCTGACCCATCTTCGATTTGTTTCCCATTCTGTAATTGGTCTACTGATTGGCTACCTATACCTGAACAAAGGCCAAGACGCCAGCAATATCATCAACAATGCTGGCTGCGTTTTTTTCACAGTAATGTTTCTTATGTTCTCATCAATGATGCCCACCATTCTCACGTTTCCATTGGAAATGGATGTTTATAAGAGGGAACATTTAAACAACTGGTATTCACTTGGACCATTATATATGGCGAAATCTCTAGTGGATATTCCGTTTCAGATGGTTTATACAATAGTCTATATCTCAATAGTCTACTACTTGACTGATCAACCTCAGGAGATAGAACGATTTTCAATGTTCTTGTTTGTGAGCATTCTTATGTCTTTGGTGGCTTCAGGCATAGGTCTTCTAACTGGGACAGCATTAGCTATAGAAACCGGCACCTACTTTGGACCCGTCTCTTGCGTACCCTGCATCCTATTCTCAGGCTTTTTTCTTTCACTGGACTCCATTCCCAAAAGCCTGCGATGGCTGGGCAACATCTTCTACCTGCGCTACGCGTTTGAAGGCAGCATGCTCTCGATCTATGGCTATGACAGACCCAAACTCGACTGCAGCGAAGTCTACTGTCATTTCAGGGTACCATCTCAATTCCTCAAACATTTAGGCATGCAGGATGCCTCCTATTGGTTCGACTGTTCGGTGCTGTTCTTTTTTATTGTGCTGCTCAGACTGGTTACCTACTTTGTGCTTAAGTATAAAATCAAAAATTTCACTCCCTAG

>*sfABCG4*

ATGAGTAGATCAGTGGCCAATAAAGTTTCTCTACACGATCCTTTGTGTTTGACGTTTCGTGACCTAACGCATGAAGTAAGATGCGGTGAGGGATTTCGTTTCATGAAAGAGACAAAATGCTTACTCAATGGAATTTCAGGCGAGTTTTATTCAGGAGAGCTCTCAGCTATAATAGGTCCATCCGGATGTGGAAAAACTACCCTCATGGACATTTTATCTGGTTACATAGAACTGAAGGCAGGGTCGATCCATTTTAATAGAGCTGAGGAGAATAGGAGAGTTCGTTGCTGCTATATAATGCAAGACGACATTTTGCAACCTACACTAACTGTTCATGAGACTATTAAATTCGCAGCTAAATTGAAAATCAAGTCAAGGAGTCTTCAGGAGAAAAAAGTGAAAGATATTTTAGATATAGTTGAGCTTTCAGGAAATTTGAAATCACTAGTCTGCAATCTATCGGGAGGTGAAATGAGAAAACTATCAATAGCTGTTGAGCTTCTCACTGAGCCTTCAGTTATGTTCCTGGATGAACCTACAAGTGGATTGGACATTTCATCGGCAGAAAAGTGCATGAGAGCATTGAAGGCCGTTGCTAGCAAAGGAGTGATGGTGGTGTGCAGCGTGCATCAGCCCAGTGGCGCCATGTGGAACTTATTCGATCACGTCTATGTGATGACAAGCGGAATGTGCACGTTCCAGGGCTGTCCAAGAAGATTGATGGACTATCTTCACTCACTTAATCTCACTTGTCCTATAAATTACAGTCCTGCTGACCATATTTTAGAAGTCACTCTTGAAGAATACGGAAATAACCTCACGCGATTGGTTGGTGCATCTAGAAATGGAGCCAGTAGAGAATGGAGACATAGACACAGACAAATCTCCAATGAAGGTGATAATGATAGAATTGATTCAGAGAAATCAACGCTTCTGAATTCTATCATCCTGGGTCAAAAATACTATGTGATTCCATTCATGATACAATTTCTTATTCTTTTGCAAAGAAGTGCATTAGGACTGATAAAATCAAAGGAAGCTATACGAATCAAGCTCATGATCCACCTGATAGTGGGAATTTCCTTTGGAATGATCTACTGGAGAATAGGGGTTGATGCATCACATGTGCGTGACAATCATAGTCTTCTTTTCTACACCGTGGCATTTATAATGTTCACAGCTTACTCAGGAATGATTCATGCCTTTCACTTGAAAGTGCGAATTACCACTCGTGAATATTTCAATGATTGGTACTCGTTGAAAGCATTCTACTTGGCCGAAAATATTGTAGACATGATCTTTCAGACATTGTGCTCAACGTCAATGTGTCTATTGGTTTACACTTTGTCAGGACAGCCGCTGGATCCCGTACGGTTTTTTCTATTCTCCTCAGCCATTGCCATGATAAGTCTTATCTCCCAAACATGGGGCATCTTGATTTGTACAGTGCTACGATTGAAGCACGCAGTAGTGTTTGGCTCTTTGTCAATCATGCCGTGGGTGGTATTTGCCGGCTACTTCCTGCGTCTAGAAGATGCGCCCTGGTTCATGCACTGGCTGTTCCAAATCAACTTCCTCAAGTACGGCTTCCAGTGCGTCATCCTATCAGTCTATGGCTACAACCGTCCTCGCTTGTCCTGCTCAAAAGATTATTGCCATTTTGTGTTTCCACAAAAATTTCTAAATCACCTTCAGTTGCAAAATGAAACATTTTCATTCAACTTTTTGATCCTGGTGACAATTCTCATTCTATCCAAGACTGTAACATTCTATGTGCTCAAATATCAATTGAAGCACAAGAGAAAAAAGCTTGATGATGAGTGA

>*sfABCG5*

ATGGTGGGCCGGCAACAAAGAGACATGGAGAGGAGGTACTCGATAGCCGAGGTGCCTTCTGAGTTGAGTGGGATGCCTCCTCCCGGCCTGATGCCTTCGGCTTCAGAGGACCTGCATGCCTGGTCTATCTATAGGCAAAACCTGAACTCAGACTTTACAGATTCGGCTCTGGGCTCAAGTGAAAAATCTCCTCTTCCATATGGCAACTTTCAACTTAGGGAATCGACTGTCCAGTCAATTCTCAGTCACCCTCGATATGGACCCAAATCAGCTTTGGGCTCCAACATGTACACCTACCTGAAGTTTGGACTTCCGCGCGTCTTCCCCCCGAACGGCGGGAGGGGCGGACGCGACGGTAGCAGCGGCTACGACAGCTCGGACGACGGTATGGGACACGGACACGGACACGGCACTGCGTCCCGGACCGGGCGTCCCGGACACGGAGCGCGGACAAGACACGCCAGTCAGCAGCATTTGCAAGCACCGTCCGGGTACTATTTAAGGGCTAGGAGTGATCCGGATTTTAGAAATACGCCGTATCATGGGCCAAGCATGCCTCTCAGACAACAGATGCCGGGTGGTCCGCCCCCTCATCACATGGGGGGTGCCGGCGGTGCCCCCCCAGGCGCCCGTGGCAAGAGTGTGAGCGAGGCCAACCTGCTGGCGCCCGAAGTGATGCTACGCCACAATGTGGCGCCCCACGAACATCGGCGCAGCGTGCATGATCTTAGGGGCGCCGCGATTGCCTACTCAGAACTGGGCGCTGCACCACATGTACTCGTGCACCCGGCTCGACATGGCGGCCGACCAGCCTCAGTTGCAGTCGTTGGTCACCACCCTCTGGCTCATCACCATGGAAGTCACTCCGTACTAGACGGGGGTGGTGGAGGGGGAGCCCCTCCCAGCATGCTCGGCATGCCTCCCCCCTCCTCGCGTGCCTCCCACTCTGTGCACCCCCCTCCACCCGGCATGTCGTTCCAGGTGCATCGCGGAGAGGCGTTTGGCGGACAGTATCCGCACTTACAGGTAAGGGGTTTGGATGTAGATGGTAAGAACAATGAACCCCTTCTTCAATCGGTATCATTCGAGGCGAAGGCTGGAGAAATATTGGCCGTTATGGCAACACAAGTGGACGAAGGCCGTGCCATCCTGGACGTGCTGTCGGGCACAAGGCGAGCAAGGACCGTTCACATCGTGCTGAATGGACAGAGCATCGGACAGCGTGTGCTGAGGAGGAGGGTGGCCTATGTTAGGAGTGACTGCACTCTGGCGGGGAGTTTGAGCGTTTCGCAGACGCTTGCTTTCTACTCGAGGCTGAGGAGACCACCGTTGGGACCCACCAAAGTGTCATCTACTGATCAGATGGACCTACTGATAGAGGAGTTGGGACTCAACCAGGTTTTGGACACAAAAGTGGCCAGTCTGACTGATTCCGAGGCGCAAAGACTGAGTCTGGCCTGTCATCTAGTCTCTGATGCTGAAATACTTCTTCTAGACCGTCCCACCCGATCTATGGATATTTTTGATACCTTCTTTCTGGTGGAGTTTTTACGGCAATGGGCCGGAGGTAGTAGTACAGGCGGCCTAGTCGGAAGAATAGTGGTGCTTACCATTCAACCGCCAACTTACGAAATTTTCACAATGGTGTCGAGAGTGCTGCTGCTATCTGGTGGCAGAATGATGTACTCGGGAAGACGCCGTGACATGTTGCCATATTTCTCAGCCGCCGACTATCCCTGTCCTGCTTTCAAAAATCCCTCCGATTATTACCTTGATTTGGTAACCCTGGACGACCTTTCGGCAGAAGCCATGCTGGAATCGTCGCAGCGCATTGAGCAGTTGGCAGAACTGTTCAGAAGGCGGCAAGAGCCTCTGTCCGATCCAGGTCCGCCACAAGCTCTTCCCGGCAAAACACGGACGGCCAATGTCTGCTCACAGACCGTTGCTTTGCTCATGAGACAACTGGTCTACTCACAGCCGACTAGTCTTACTAATTGGTTGACCCATGTACTTCTAGCTGCCATACTATCGCTAATTGTTGGTGCTATATTTTGGGACGTGCCCAAGTCGGATCCACAACTGCTCTATGCCGACAGGATGGGATTTCATTACACAATGATGTGTGTTGCATCTCTACCGATTCTTCTCATGTTAACTCTAAACGATGCTCGTAGCTCAGAGAGAGCTGCTTCTCAAATGGATATTAGGGATGGACTCTATTCTAAGCTTATTTTCATCATAATCACTGCCCTTATCAGTTTTCCAGCCGTTCTGTTCGTTTGGCTGGCTTATATAATTCCAGCCTATGCGATGACCTCATTATATAACCAGGGTTCACAAGCGCCCAACGGATTCCACATTTATATCAGCACAATGCTAGTCCACATGATGTGTCTCTACTACATTCTGCGTCTGGTCACACAAATCTTCCATTCTCGACGTACATCCGCCATCATTTCCGGTCTGGTACTCTTCATATTTTCTCTCGTTTCCGGTTACCCCATCTACCTTCCCGATGTCCCCAACTGGCAATCCAACTACTTCGGACTGGTATCACCTGTCAGATGGAGCATGCCTGGTCTGCTGGCTAGGGAGTATTCAGGTGTTACGTTGGCAGCCATTGCTTCGCAGATGATCTGCAATAATCGACAGGTCCAGCAACAAGACATAATCGTCCAACTACCCTGCCCCATACCAAACGGGACAGCTGCTCTCTCCTTCTACGGCCTGAGTCCAAAATCGTCGGTACCCTTCAACTGGACCCACATTGTGCCCTACTGGCCCCCCGTAATTATTGCACTTGTTATGGCCGTCCTCCATATCGCCATTTTCCTTTTCAGATCACCCACGCCCGCTTGGAAAAAAGAGGATAAACTGAAGAGATACATTTATCATCCTCATTGA

>*sfABCG6*

ATGGAACTAGAAACAGAGCTGAGGTCCACCTCAGCGGGAACCTATTTTTCGAGGCATTCAACTAGAGAGCCAGTTGAGCTGAGCTTTGACAACCTCACCTACTCTGTTTCACAGGGATTTCGAAAAGGCACAAAGACTATATTACACAACATTGGAGGTAGATTCGAGTCTGGTCAAATAATTGCAATTATGGGACCTTCTGGAGCTGGAAAATCATCTCTGCTAGATCTGCTCTCAGGATACAGAATATCAGGTGTAGCCGGATCTGTGTACGTCAATGACCGATTTCGAGACCTTGATGAATTCCGAAGGCTCTCCTGTTATATCCAGCAGGACGATCGACTACAGCCTTTGCTGACTATTGATGAGAACATGTGGGCAGCCGCTGACCTAAAACTGCCTTCAAGTGTTACCACCAAGGAGAAAAGAGCTATCATAGACGAGATTTTAGAGACCTTGAAACTCTCGGGCTCTAAAAAGACCAGAGCTGGACAACTTTCAGGAGGACAAAAAAAGAGACTTTCAATCGCATTAGAACTAGTCAATAATCCATTAGTAATGTTCCTGGATGAGCCAACTACAGGTTTGGATAGCTCCTCATGTATGCAGTGTGTAACACTGTTGAAAGAGCTGGCATCCCAAGGCAGAACAATAGTGTGCACCATACAtCAGCCGAGTGCGTCCCTGTTCATGAAATTCGACCACGTTTATGTGTTGGCGGGTGGACGGTGTCTCTACCAGGGGTCTTCCGGCAATCTTGTTCCATATCTGTCTGAATTGTCGTTACCGTGTCCGACCTACCACAATCCAGCCGATTATATTATCGAATTGGCCTGCGGTGAACATGGAGAAGACAAAATCGAAAAACTTGTGGATGGAACAGAGAACGGAAAATGCTATAAATGGTTCACAAACGGAGAAGTAATAAAATACAACAATAATGCCGCAGCTGACGTCAAATCCATGGGTTGTTTGCCTATCATGAAGAAGTGTGGAGGCTCACTCCAGGTCACATCTCAATGGAATCAGATCGGCGTCCTACTACGGAGAGGATTCATTAAAATGAAAAGAGATCAGACTCTCACTCACATGCGATTTATGGTGAATGTGCTAACTGGGATGATGTTGGGATCGCTCTTCTTCCAAACTGGCAACAAAGGAGAGAGAGTTTTAGATAACTTCAATCTGTTGTTTTCAATATTGATACATCACACAATGACCACCAAAGTCCTCACCATCCTCACATTTCCAATGGAGATGTCCATATTGAATAAAGAATATTTTAATAGATGGTATTCATTGAAGTCTTACTACATTGCAACAAATATTCTGGACATACCAGTTCTGACTATTTGCGCTATAACGTTCTCAGCAATAATCTACGCAATGAGTGGTCAGCCTTTGGACTGGACGCGATTCAGTATGTTCACTGGCATAAGTTTATTGGTGGTTTATATTTCACAAAGTCTTGGCTTCATGGTTGGATCTATATTCAATGTTGTGAACGGAACGTTTGTCGGCCCAACAATGCTCGTTCCCATGATGATGTTTTCTGGGTTTGGCGTTTCGCTGCGTGACATTCCCGAGTACATGAAATGGGGAACAAATCTCAGCTACTTGCGCTACAGTCTTGAGGGATATGTGGCCGCTATCTACGGCCTGGACCGACCCATCCTGCCCTGCTCCAGTTACTACTGTCATTACAAATACCCGAAAAAGTTTATGTCAGAGGTTGCCATGAATGGAGACCAGTTCTGGATGGATGTTTATGCTCTCCTTTTCACTTTATTCCTGACAAGGGTAGCGGCTTACGTTCTACTCAGATGGAGGATAAGAGCTATGAGATAA

>*sfABCG7*

ATGAAGTTTATCGATGAAAATGAGTCGTGTGAAATGAACCAGATTGTTTCTTACGACAGGAAGGGCTCGGTGAAGGTGAACATCCAGCCATGCCAGCAGGCGCGTACCCTCACCCACCTGCCCAAGCGGCCTCCCGTCGACATCGCCTTCGAGGACCTCAACTACTCCGTGTCCGAGGGCAGGAAAAAGAAAACAAAAACGATCTTGAAATCTGTGAGTGGCAGACTCTATTCTGGAGAACTGACAGCGATCATGGGCCCCTCTGGGGCCGGAAAATCAACTCTACTCAACATTTTGACTGGTTACAAAACCTCTGGAATGAAGGGTTCAATCAAAATCAACGGCGAAGAGCGAAATCTGAGCCAGTTCGCCAAACTGTCGGCCTACATCATGCAAGACAACCAACTGCACGGAAACCTCTATGTGGAAGAAGCCATGCATGTAGCAGCCAACTTGAAATTGGGCAACGAGCACAGCAAGGAAGAAAAATTGGATGTGATTCAGGAAATCCTGGAAACATTGGGATTACAAGAACACAGACGCACGCTGACTTGCAACCTATCTGGAGGACAAAAGAAACGTCTCTCTATAGCTCTTGAATTGGTCAATAACCCTCCCATCATGTTTTTCGATGAGCCAACTAGTGGCCTGGACAGTTCGTCGTGTTTCCAGTGTGTGGCGCTGTTGAAGTCGCTGGCCACCGAGGGACGTACCATCATCTGCACCATTCACCAGCCGAGTGCGCGTCTCTTTGAGATGTTCGACCACCTGTACACCTTGGCTGACGGGCAGTGCGTCTACCAGGGCTCCACCGCACAACTGGTGCCCTGGCTCAAGACCCTCAACCTGGTGTGTCCCAGCTACCACAATCCAGCTTCGTTCGTTATCGAGGTATCCTGCGGTGAACATGGTGAAAATGTTAAGAAACTAGTTGCTGCTATCAACAATGGCAAGAACGACATCAGAACTGGCAAGCCGTTCCCGAAGCTTGACTTCGCAGCACTGAACAATGCCAGCATGGAGAAACAGTCCAACTTGAGTCAGGAAACTGACAATTTGCTGGCCAATGGAGATGCTACATCCGACCAGCCACTGTCTGAGGGCAGCAACTGCACCAATAATATGTTGTTGGCTTATGCCACCAATGATATTGCTAAGGATTCTCAATCGAATTCCGATGGGAAAGGAGGAGTGGTGATTCCAGTGGATTTCAGTGACAACGAGAAGAACAAGCAAGACAACGTTTCCACGAGTCTGTTGGAGACGACGCTGCCGCTGTCGCAAAAAAGATACGGCACCTCGGAATTCAATCAGTTCTGGATTGTTTTGAAGAGAACTCTGCTATTCTCACGCAGAGATTGGACACTCATGTACTTGAGATTGTTTGCTCACATTCTGGTTGGATTTCTGATCGGAGCTCTCTACTATGACATTGGAAATGACGGAGCCAAAGTACTCAGCAACCTTGGATTCTTGTTCTTCAACATGTTGTTCCTTATGTATACGTCGATGACTATCACAATTCTCTCTTTCCCTCTTGAGATGCCTGTATTGATAAAAGAAAACTTCAATAGATGGTATTCGTTACGATCCTATTATCTGGCCATAACAGTTTCCGACATACCATTCCAGGCCGTGTTTTGCGTTTTGTACGTGTCAATAGTGTACTATTTGACGTCGCAACCGCAGGATATGACACGTTTTGGAATGTTTTTGGGTGCTTGCCTGCTCATCTCGTTTGTGGCACAGAGCGTTGGACTAGTAGTGGGAGCGGCTATGAATGTTCAGAACGGTGTATTCCTGGCTCCGGTGATGTCTGTACCGTTCCTGCTCTTTTCTGGATTCTTTGTCAGCTTCGACGCTATACCGGTCTATTTAAGATGGATCACGTACCTCAGTTACATCAGATATGGATTTGAGGGAACTGCACTAGCCACCTACAGCTTCAACCGAACAAATCTTAAATGTTTCCAGGTATACTGTCACTTCAAGGACCCAAACACCACATTGGAGGAACTGGACATGAAGACTGCTAGCTTTCAACTAGACATCATCGCTCTAATTGTCATATTTTTCTTCTTGAGAATCTCAGCTTACTTGTTCCTCAGGTGGAAACTGATGTCATCCCGTTAA

>*sfABCG8*

ATGGAAGGAATCAACGCGTTGAATAAAGCGAAACACAATATGGAATTTTACAATAACAATGACTACGAACAGTTTAGCTTCCCAAAACGACCTACAGTGGATATCAATTTCCAGGACATCACGTATACTGTTAACACAATTACAGCAAAAAAAGAAATTCTCCACGGCGTTAGTGGAGAGTTCAGGTCTGGGCAGCTGACGGCCATCATGGGACCATCAGGCGCGGGAAAAAGCACTCTGTTGAACATTCTGGCTGGATATACGTTAAAAGGCTGTCAAGGAAGTATATGCATAAATGGCATCAACAGAAAAAGTCGAATTGAGCAATTCCTGAAGATGTCCTGCTATATTACACAAGATGACGAGCTGAGACCTCTCTTGACTGTCAGAGAAGCTATGATGCTGGCGGCTCATCTCAAACTTGGCTTCACTCGATCCAATGCTGAAAAATCCAAACAGGTATCTTACATACTGGGTCTTCTTGGTTTGAAGAAACATGAAAACACCAAAACCTGTCGATTATCTGGCGGACAACGAAAGAGGCTATCAATTGCTCTTGAGCTGCTCACAAATCCGCCGATCCTGTTCCTTGATGAGCCCACGACAGGGCTAGACAGTGTGTCAACCACGTCCTGTGTTTCGCTATTGAAGAACCTGGCGGGTGAAGGGCACACAATCGTTTGCACGATCCACCAACCGACCGCTTCCATGTTTGAAATGTTCGACCATCTCTATGCGATAGCTGACGGTGATTGCATCTACCAGGGCTCGAGTAGTAATCTGCTTCCGTTTTTGTCCTCGCTAAGTCTACATTGTCCCAAGTACCACAATCCGGCTGACTTTTTGATTGAGGTAGCTGTTGGGGAGTATGACTCAAATATAAAGTCTATAGCCGCGGCAGCTGCTAAGCATGGTCGTCAAGAGACCACACCTTATTCTGAGGAGATAGTCAAAGAGGAAAATGGTCTACCCGTACGAGTATTGCAAACTAAGAAGTCGCCAGCTTTTGACATTATTGAGTACACCTCCTGCTTAGCAGAGCCACCTCCATTTTGGTATCAAGTATTTCATCTTCTCCATAGAAACTTTATCATCACACGTAGGTCTAAGTTGTCCCTTGCCTTAAGAATGTTCATGCACATTGTGATCTCAGTAATGTTTGGAATCATTTACAATAATGTTGGAAACAACGCCAACTCTGTGTTTGGAAATTACATTTACGTTTATGGAACAAACCTGTTTCTACACTATACCGGGCAAATGGCAGTCACTTTATCATTCCCATTGGAATTCAGAGTCTTGAGAAGAGAGCATTTCAATAGATGGTATTCCTTATTGCCTTACTGCGTAGCAACGCTGCTTATCGAAATACCGTTCCAGATTATGTGTGTCATTGTCTACCTAGTGCCCAGTTACCTATTGACTGGGCAGCCCTTGGAATGGATCAGATTTTTGATGTTCTTGATGTTCACGGTAGCTGTTTGTCTAACTGCTCAAGCTTTTGGATTTTTAGTTGGAGCCACAACTCCTGTCACGTTGGCTGTCTTTATTGGACCAGTGATCACGGCTTTCCTGTCAGTGTTTGGATTCGCAATGAAGTACAATGACATTCCTTCATACCTTCGAGTTTTCTATCACATATCGTACTTCAGATCGTCTTTCCAGGGGTCGTTGATGAGCTTGTATGGAAATAACCGCTCCTATCTGCCGTGCCTTGAGAACGGCTTCCACGGACGCAACGGCTACTGTCATTACACACATCCCACAAAATTCCTCAGAGAAATGGAATTCGAGGAGCCAAACCCAGTTTTTGATGTAAGCTACATTGTTAGTGTTTGTTTATTGGTGTATATGTTTACAGCGACTGCTATTTGGTATAGACTTAACAAAAGGTGA

>*sfABCG9*

ATGGACGTGGAATTCGAGGATTTGACGGTCAGAGTGAAAAGCAGTTGGTTTCAGAGAGAATCTGGCAGGAGGATTTTGAAAGGAGTCAGTGGAAAGTTCAAAGCTGGACAACTTTCAGCCATACTTGGTCCCTCAGGGGCGGGAAAGAGCTCCTTGCTCAATGCAATATCTGGTTACCGGTCGGAAGGCGTCAGCGGTCGTTTAAAGCTGAATGGAGTGCATCGAGATGAGGCAAGGTTCAGGAAGATGTCCTGCTACATTCAGCAGGAGGATCTTCTACAACCTATGCTTACACTGCAGGAAGTTATGAACTTTGCCGCCCTACTCAAACTTCCCCCGGGAACTGGGTATAAGCAGAGAAGAGCTGTGATAAGCGATATACAAGGAATCCTGGGTTTGATTGAGTGCAGACACACGCGTACTGAGGCATTGTCCGGAGGCCAGAAGAAAAGACTATCAATCGCCCTTGAACTCATCAACAACCCTCCTGTCTTATTTCTAGACGAACCTACCAGCGGTCTGGACAACGTCTCCACGTCCTACACACTGAAACTGCTAAGCACGCTGGCGCATCAGGGCCGAACAATAGTGTGCACCATCCACCAGCCGAGCGCCTCCCTCTTCCAGATGTTCGACCACGTCTACGTGCTGGCGGCCGGCCTCTGTGTCTATCAGGGCGTCACCGGCGAACTGGTTCCCTTTCTGTCTTCAGTCGGACTACACTGTCCAAGGCACTACAATCCGGCTGATTTTGTGATCGAAATGACTGATGGAGAAGACGAAGATAACATCAAAAGGTTATCTTCAGCGATCAAAAATGGGAAGGTTGTCCAGCTAACGCCTGCTGATGCGAAAAAATCGATTCCAGATTTTCCTCAACTACCAATAGAAGGATTACCCATGGAAAGACTACCCATAGGAGGACTACCATTAGAAGAGAAATTAGTTGCAGTCAGTGGCTATGGTGAGAAATACGTTGACATGGACAATGGAATCTGTCTCACTTGTAGGGCAGACTCGTCTGCTTGGCTAGAATTTTGTACGCTTTTTAGAAGGATGTTCCTTCAAATTATGAGAAATAAGACTGGTTTAAAAATTCAATTTTACCATCACTTGGTGTGCAGTTTGGCAGTTGGAATAGTGTTTTGGGGCAAGGCTCGTGACGGTAACCAATTTTTCAATCACATGAAATTCTGCATGGGTATCATCCTCTTCCATGCCTATACACAGTGTATGGTACCTGTTCTTACCTTTCCATTCGAAGTTAAACTGCTGAAGAAAGAACACTTCAATCGATGGTATCGACTAACGCCGTATTACATGGCATTACAGCTCTCTAAAGTTCCAACCATGACACTATTCAGCCAGCTATTTCTTACAATAGTTTATGTGATGTCAGGCCTGCCGCTCGAGTTCTACAGGTTTTTTGTCTTCTCAGTCGTTGGCGTGATGACTGCTTTTGTAGCTGAAGGGTGGGGTCTGGCTATTGGATCAGTATTTAATGTCACAAACGGCAGCGCAGTCGGCCCCATGACAATCGCTCCGTTCCTCGGCTTCGCCATCTACGGATTCGACTTCGCTCGCAGCATCCCCGCCTGGTTCATGCCCATACTCAAGCTCAGCTTTTTGCGTTCGGGTGTGATCGCACTGATCATTGTTGTGTTCGGCATGAATCGTGGACTCCTCGACTGCAATCACGAGATGTACTGTCACTTCAAGAATCCGCGAATCATAATTTATTACCTGGATATAGAGGGTGTCTCGCCGTGGCAGGAGATAGCTGGCATGTTCGGTATGCTACTATTCTTCCGGATAGTTTGCTATATTGGACTTAAATGGCGGCTCAGAACATGA

>*sfABCG10*

ATGATAGGTAACGATTATAGTTTGGAGCTGTGTAATATATTTCACACAGGGCAGGTTGAACCAGGCTCATGTTTACAAAGGATATTTGGCAGTGTTCAAACGGGGCTTATTCTGAAAGATGTTTCTCTCGAAGTTAGAGCCGGAGAAGTTCTTGCAGTGCTTGGATCAAAAGGCAGCGGTAAAAGAGCTCTACTAGAAGTGATATCTAGAAGGAGCCGTGGTCCCACCAGAGGCCAAATCCTCCTGGATGGTGCTCCCATGACCTTGAGTTTGTATCAGAAGAACTGCGGCTATGTCAGCCATCGAGTTGATCTAATTCCTTCACTCAATGTGGAGCAGACTCTACATTACGCGGCTAATCTCACCATTGGATCACAGGTATCCAGGTACGTGAGGAGTTCCCGTGTGCGTCAGGTGCTGGCTGATTTGGCTCTGAGTCAGGTGGCAAGACGCAGTGTATCCAGTCTCACACTCAGCGAATACAGGCGATTGGCTATTGGAATACAACTTGTCAAGGATCCAGTTCTACTCCTCCTGGACGAGCCAACCGCCAACCTAGACCCCCTTTCCACCTACCTGATAGTCTCCATGCTATCTTCGCATGCCAGACGCCGCGGCAGAGCAGTAGTGCTGACTATGGAAAAACCGCGCTCCGATGTCTTCCCATTTCTGGACCGAGCCGCGTATTTGTGTCTGGGAGATCTGGTGTATGCGGGACCCACTCGTCTCATGCTGGAGTACTTCAGAGCTATCGGCTTCCCCTGCCCGGACCTGGAGAATCCTCTTATGTACTATTTATGCTTATCAACAGTGGACCGGAGGTCTAGGGAGCGTTTCATTGAATCCAACACACAGATCATAGCTCTGGTAGAAAAATTCAAACTGGAGGGAGGACCTTACAGGAAGTCTTCGGCAGGAGGTGGAGGAGGGGGACATGTGCTGCTGGGATCAGGGGAGTCACCCCCCTCTCATAAAATGCCCCTCACCACCCTAGGCAAGCCAGGAGCGATTCAGCTTGGGTTCACACTCTACCAGCGACTGCTAGCTTCCACCTTCAACCTATCAGCAATCGCCGCCCAGCATCTCTTCCTTCACCTCGCCCTCTTCCCCCTCATCTGCACGCTTATCTGGTTCTTCTACCGCGACGTCAAACACCAGGACGGACCCTACACCTTCCAATCTCTCAACGGATTCCTTCTGAACAGTCTGCTCACCTCCTGCGCATGCGCCATTGTCAAAACCGCATGCGTCTTTCCCATACACCGCACTCGTTACTACCAGGAAGCTCATGAAGGGCTTTACTCGGGACCGTTGTTCTTACTAAGTTACAACCTTTACTCCCTACCTTTTTCAATACTAACAGTAGCTATTGGTTCTCGAATACTTTTCGAATCAACTGGTCTAACTTCCAGTACAGATTGGTTCTTTTTTGCAGCAATACTACTTTCCACCTACTTACTATCAGAACAACAAACTGTCGCACTTTTAATGATAATCAAAGGATCCTTCATTGCATCAATCACTAGTTTGTACCTAGGTACAATATTTATCATACTAAGCAGCGGTACCCTAAGATCCTACGCTAGTTTACCGGAGTGGTTGCTGTATCTAACATACGCGTCTCAAACGCGTTACTCCAGCGCGTTTTTGTCGCGTCAGCTGTTCGGTTCAGTGTATACCGCGTTACCTGGTAATTGTACCGCGCGTTTGCCGCTGAACGACGCGTTTTTATGTCGCTATAAGGATAGCACGGCGTATTTGGCGGAGCGTTATAGTAGGGGGAGTACGGTGTTCAATATCAATGATATGTTGGATAGTGATTTTAATTTGAGTTTTTCGTATGCTTTCCCTGTGGGATTTGTTTTGTTGAACTGCATATTGTATTTGATACCTTTGCCCTCGTTTATTAAAGCTAAGTTCAGGGATTGA

>*sfABCG11*

ATGACGGAAGAACGTGCTGTACTGCTGCATTTGCCTCCCAGCAAACCTATCCACATTTCTTTCACCGATATCACTTTGACGGTTGAGTTGGGCACTGTACGAAAAACCAGGAAACAAGTCCTCAAAGGGCTTGCAGGCAGTTTCAATTCTGGAGAGTTGACAGCTATTATGGGTCCATCAGGAGCTGGAAAATCTTCGCTACTCAACATCCTAACCGGCTTTCAAAAACAAGGCATGACCGGAACAATAACAACAAGTGGAGCTGGAAAAATAGAAAACTATTTTAAAAATGGAGTGAACACAAAACAGTCATGTTATATTATGCAAGATGATCAACTGAACCCACTCTTCTCTGTATTTGAAATCATGTCAATGGCAACAGATTTAAAATTGAGTCCAGCTATTTCACAAAAATCAAAAATACTCATTATTGATGATATTTTAGAGACCATTGGTCTGATGGGTTGTAAATACACACGGTGTGGAAGATTATCAGGAGGCCAGAAGAAAAGGTTATCCATAGCTCTAGAACTTGTTGACAACCCTCCAATCATGTTTCTTGATGAACCTACCACGGGACTGGACAGCTCCAGCACCGTTCAACTGGTCTCTCTACTGAAATGCTTAGCAAGGGGCGGAAGAAACATAATTTGCACAATCCATCAACCCAGTGCAACCATTTTCGAAATGTTCGATCATGTTTACTTGATCAACGGCGGACGATGTGTTTATCAGGGCTCAAGCATAAATTTGGTCAAGTTCCTGCAGTCAATCAACATTCCATGTCCCAAGTACCATAATCCAGCCGATTTTGTGATGGATGTTATTAGTGGCGAGTTTGGTGATCACACAGACAGAATGATTGAAGCGTCCCAAAATTCCAATTGGAGAGCTCCACCACCTGTAATCAGAGCGCCATTCAAAAAGCGAACATCAGATGACATTGAAAAAGTGAAAATGATGGGAGTTCCAGTTACTCCTCCAGAGATTCTCAGGTTATGGGTCCTTATCAATCGATGTATCATTCAACTCTACAGAGATTGGACAGTAACACACTTAAAAATGATAATGCATTTTGTAGTTGGTGTAGTCATGGGCCTAATTTTCAACAAGTGCGGTAACGATGGTAGTCTTAGTGTGAATAATATCGGCTTCTTCCTATGTACAAACGTGTATTTGAGCTACACTTCAATAATGCCAGCAATTTTGAAATTTCCATCAGAGCTCCACATACTGAAGAAAGAACAGTTCAACAACTGGTATAAGCTCTCCACCTATTACATCGCATTTCTACTCACAAATATTCCTGTACAGATGATGTTATGCACCGTGTATGTGTCAGTTTCGTATTACCTCACACATCAGATTCAAGAATGGCCACGATTCGCCATGTTCTTGGCTGTCAATCAATTTTCAGTCGTTATTTCAGAGTGCATCGGTCTAGCACTTGGAACAACTATCAATCCGGTGAACGGATTATTCACAGGCTCTGTACTATTCTGTTTTATGCTGTTATTTGGAGGATTCTTGGCTCTCTACAAGCACATGACTTTGCCGCTCTACTTAGTCTCGTTTCTAAGCTACATGCGCTACACAATGGAGGGAATGGTGCTCTCAACATATGGCTTCCAAAGACCACTACTAGACTGTCCAAAGAACTACTGTCATTACAGAATACCCTCTGTCGTTTTAGAGGAAGTGGACATGAAAGAGGATCATTATTGGATAGATGTGATCATTCTAATTAGTATGAGCGTATTCTTCTCATTTTATGCGTATATCACCCTCAAGCGTCGCGTTATGCATAGGTAA

>*sfABCG12*

ATGTGTGCCAAACGTCATGGACAAGTTACCATCGACTTTGACAATTTGTCTTACAGTGTACCTGAGACAAATAAAAAATGTCCACACCGAGTGCTTCTTCATAATGTAAGTGGACATTTTCGTCCAAAGCATTTAGTGGCCTTGATAGGACCTTCGGGAGCTGGCAAAACAACACTTCTCAATGTAATATCTGGATCAAAGGCAGTGGATAATTCACGAGTATCCGGTAGTATACTTGTCAACGGAAAAGAGCGAAATTTGCAAAAATTTCGAAAACAGTCGTGCTACATCACGCAGGAGTGGTCTCTCCTGAATCAGCTGACTGTTGAGGAAACTTTGGAAATTGCCGCTCGATTCAAGTTGCCAAGTAATACCAGTGAAAGCGATCGGAAAAGTACAATCAACGAGGTGGTTGAAATTTTGAGACTGAATGGATCTAAGAACACTCTAGTCAAAAATCTCTCGAATGGCCAGAAGAAACGAATTTCTATCGGAGTTGAACTCATGAATAATCCACCTGTCTTGTTCGTTGATGAACCCACAAGTGGTTTGGACAGTTCTTCAGCACTGCAAGTGGTCAGCCATCTACAGAGCCTAGCACTGGATGGCCGAACTGTGATAGTGGTCATACATCAGCCGAGCTCCAAAGTTTTCCAACTTTTTCACGACGTTTACTTGCTGTCTGACGGCGAGTGTCTCTATAATGGTCCCTCGGAGCATTTGGTTACTGCCCTGTCGTCGGCTGGCTTCAACTGTCCACAGTATTACAGCAAGTCTGATTTCGCTATTGAAGTCGCCAGCGCAGAGGTGGAAGGAGATATAAAATTATTGAAAATGGAAACGAGGAAACGTTACGAAATGAAGGAGGGAGAATTCTATGAAAATTTAGTCCCTAGCAAGCGAATTTCTGAAGCAAATCATCCCAATGAGCACACAACAATGATGATTGGTGATGATGATGACGACGATTCCTATAATTCGTTGAAAGGCTACCCAGTTTCGAAGCTTGAACAGTTCTGGATACTTTTTATGAGATGTACAAAAATCACAAACAGAGATATGTATCTATCACGGACACGAATCATCACGCATATTATGCTGGGTTTACTGCTCGGTTGTCTCTTCTATAACTTTGGAAATGACGCGGATAAAGTAGTAGGCAACCATTCCTTCTTTTTCTTCGCAGTTCTGTTCATCCATTTCAGCAGTAGCATGCCAGCTATAATGACATTCCCATCTGAAGCCAACGTTTTTCTCAGGGAGCATTCCAACAACTGGTATTCACTGACAGTCTACTTCTTTGCAAAAGTATTGGCGGATCTACCTTTGCAGATAATTTGTCCGACATTGTTCTTGACGATTGGCTACTATATGACCGGTCAGCCAATGGAGTTGCAGAGGTTCTTCATGATTTGGTTTGTGATGATTCTTTTGGCCATGCTGGGACAATCTTCCGGCAATGCAGCTGGCGCTTTGCTCAACGTTGAGCTGAGCATCGTAGTAGTGCCAACAATTTCGATGCCTCTGTTCCTGCTGTCAGGCTTCTTTCTGCAGCCGAAAGATCTCTCGCCGCTGTTCAAGTCATTGAGCTGTGTCAGCTATTTTAAGTACGCTTTCGAGGCGGTCGCCGTATCCGCCTTCGGCTACGATCGCGGCCGGCTGCCCTGCTCGCAGCCGTACTGCCACTACCGCAACCCGCGCAAGTTCCTCGACGACATCGGCGTGGACGACTATCACTATTTGGAGCGAGTCGGCATGGTGGTGGCCTGGGCTCTTGCCACGCAATTGCTACTCTACACAACGCTCACCATCAAAATCTATCAAGATAAGATTAGGACTGCTTTCAGAAAGCTGGTGACGTGA

>*sfABCG13*

ATGATCATTTCCAAGGATCTGTTCCTGCTGGGCGACCAAGACTATCTGCGTCTTCCCAAGGACGAGAAACGGACCATGGTGCGGCCGACATCCAATAAAACTCAAAGGGTGGAATCGCATTTACCATGTCTTCAGCATCATCACCTGCCTCACAGACCCCTGGAGCTGGTCTTCACCAATGTATCCTACGTTGTGGACAAAAAAGCCATCCTGAAAGATATCAGTGGCGTTGTCAAACCAGGAGAACTTCTAGCCGTGATGGGCCCTTCAGGCTGCGGCAAAACCACGCTTCTGAACTGCCTGGCAGGCCGATTAAAGCTGGACTCTGGAAATATTCGTCTCAACAAAGAGCGCCTCAACAAACGCTGGAAGAGGCGGATATGCTACGTTTTACAGCAAGACATTTTCTTCCCTGATCTCACCCTTCGCCAGACTCTAGAGTATGCAGCAATGTTACGGTTACCCGACTCACTGTCCCACGCCCAAAAAATGCAATATGTTGATCACATAATTGACGTACTGGACCTCACAAATTGCCAAGAAACAATTATAGGAGACTACATAAAAAGAGGACTGTCAGGGGGAGAGAAAAAGCGTGCTAATATTGCCTGTGAACTATTAACAAACCCTTCATTGATGCTTCTTGATGAGCCCACGTCAGGACTGGATTCACATTCTGCCTACAATCTAATGTTATCGTTGAAAAAGTATGCAGAAAAAGAAGGAAAAACCGTTGTGGTAACTGTTCATCAACCCTCATCTCAAATATTCCATATGTTCGACAGACTACTACTACTTTGCAATGGAGAGACTGCCTATTTTGGAGATGTAAATAAAGTTGTTGACTTTTTCAACAATGTTGGACTGACAATGATGCCTCATTACAACCCTGCCGATTTTATTTTGGAACAAGTCAAAGGAAGTGAAGAGATGAAGGAGAAAATAATCACAGCAGCTAGAGAAGCCAGATTTCGACCCAACTACCCACAAGAACTGATGCCCGAATATTTCAACCAGTCCATGTATCTAAATAACTATCACGAAAGTCATTTACATTCTAACGGTCACATAGGAGGAGTGCGTTGCCAATGCCAGCGAGAATTATGGAACAATTCAAGGCATTCCACGTCACAATCTCTTCCCTCGTCAGACATGTGCGTTCCGGTGGCAGTGGCCATTGGCGAAACCACTGAATCACAGGGACACGTTTATACAACTATTGCAGTGAAAGAAGAAGAGGGCAAAACGTTATGGCAGGATACGGCCAGTCACGCGTCTTCTTCCGTGAGCAGTTCCGACGACGACGTGTCATGGCAGTGGCCTACCTGCTTCTGGACACAATTCAAAGTACTGAGCAGAAGGAATTTCCAAGAGGCGCGACCCAGGATGCTGTCCACACTGAACTGGGTGCAGACAGTGGCGCTGGGCGTGATGGCCGGTCTGCTCTGGTTCCAGCTGGAGCGCAAGGAGGAGTCACTGCATGACATACAGGGCTGGATGTTCTTCTCCACCACCTACTGGATGCTGTTCGCGCATTTCGGCGCTCTCTCCTCCTTTCCACCTGAGAGAGAAGTCATCAACAAAGAACGTCTATCTGGTGCCTATCGACTGTCAGCCTACTACTTGGCGAAAATGGTGGGAGAACTGCCTCTGACAATTACGCTGCCCGCCGTGTATCACATCATATCCTACCCTATGCTGGGCTTCCACAGTCCGACAGTCTTTTTTACACTGTTGGGATTCCTGCTTCTCAACACTATTGTAGCTCAGAGTGTCGGATTCTTCGTTGGCGCTTGCTGCATGGACATGCAGGTGTCGATTACAATCAGCGCCCTCTACACTCTGGCGACACAACTGTTCGGCGGCTACCTGGCCACCAATATTCCGCCGTGGCTCAAGTGGATGCAGTACCTGTCCATGGTGCACTACGCCTATCAGAACATGCAGATTGTCGAGTTCAGCGAAGGAGAGCAGATCAAATGCGGTCCGCAGTCCAAGTTCGACGTGTGCATCAACAACTCGACCAGCCACATTCCAGTCACGTCGATTCTGGAGGTGCAGGGCGCCAGTCTGCCGCTCTGGGCCAACACCCTCGTCCTGCTGCTCTTCCTGCTCATATTCCGCGTGCTCGGCTACATCGTGCTCAGATACTTCCGTCGGCCCAAGTGA

>*sfABCG14*

ATGTCTCGAAAAGCCGAGAACCAGCCTTTGTTAGTACAGGCTAAACATAAAACGAACGGATCTACGAAACCTTCGTATCAAACTATATCTTACGACTCATCAGCCCCCCAACAACCCGACACCTGCGCTCTCCACCTCGACAACCTCCAACAAAAGTCCCCCCCACCCACCAATGGCTACCAGAGCATCTACCCGCGACTAAATGGCGGTGACAAGGGTGGAGGAGGGGGAGGTGGCTCACCCCCCAGCACACCCTCTGCGTTCCCCCTGGAGAACATCACCTACAGTTGGCACAACATCAATGTGTTCACGTCGAGCAGGGAGTCGAGGAGGTCGAGGATGGTCAACTGTGTCAGGAGTATGGTGAGCCGCAATGAAGGCTACAGCCGGGTGGGGCATAGGAAGCATATTTTGAATAATGTTACCGGCGTAGCCTATCCTGGAGAACTGATCGCCCTCATGGGTTCCAGTGGCGCTGGAAAAACCACCCTGCTGAACAGTCTGACCTTCAGGAACTCTCCAGAACTTCTAGTGTCCGGGCAACGCGCCATCAACGGCATTCCAGTAACCTCCAATACCCTGGCATCGCTGTCAGCCTACGTACAACAGGACGATCTTTTCGTGGGAACCCTAACTGTTAGGGAGCATCTTGTGTTCCAGGCTTTGGTGAGGATGGACAGACATCTGAGCTATGAACAAAGGATGGACCGTGTTGAAGAAGTCATTGAAGAATTAATGCTTACCAGTTGTCAGAACACAATAATTGGTGTACCGGGAAAAATCAAAGGAATCTCAGGAGGAGAAATGAAGAGACTGTCTTTCGCATCCGAGGTCCTCACCAACCCACCCCTACTCTTCTGCGATGAACCCACCTCCGGTCTGGACTCCTATATGGCTCAAAATGTTGTCAGCGTCCTGAAATCCTTGGCTAATAAGGGTAAAACTCTGATCTGTACCATCCATCAACCTTCCAGTGAAGTGTACGCCATGTTCGACAAGATCCTCCTGATGGCTGAGGGAAGAGTCGCATTCCTGGGATCACTGGATCAGGCCACTGATTTCTTTAGGACTTTGGGAGCAGGATGTCCCAGCCACTACAATCCCGCCGATTTCTTCATTCAACTTCTGGCCGTTGTACCCAATAGCGAAGAATCCTGCCGCAATATGATTGAACTGGTTTGCGATACCTTTGCTACTTCCGAGATTGGTACCAAGTTGGCTTTGGAGGCAGAACCACGGGTACCTGATCATAAGTCGAAATCCATGTTTGTTGGTGCATGGGGTGATACTCTTTACCCAAGGGCGCTCTCGCCTTACAAAGCATCATGGTACAACCAATTCAAAGCCGTATTTTGGAGATCATGGATCAGCGTTTGGAAGGAACCGGTTCTGATCAAAGTCAGGATGTTACAGACTTTTATGGTTGCACTGATGGTTGGCATAATCTACTTTGGACAGGAGAACGACCAGGATGGAGTAATGAACATAAATGGAGCTCTCTTTATCTGTATCACCAATATGACTTTCCAGAATGTGTTTGCTGTTATTAGTGTCTTCTGCGCCGAGCTACCTGTCTTCCTGAGGGAACATTTCAACGGGATGTATCGAACAGACGTGTATTTCCTTTGTAAAACTCTGGCCGAAGTCCCTATATTCTTGGCGATACCTGTACTATTCACTGTAGTCATGTACTATATGGTGGGCTTGAATCCGGCTCCATCAAAATTCGTACAGGCCGGAATTATTATTACTCTTGTTAGTAATGTTGCTACATCTTTTGGTTATCTAATATCATGTATCAGCTCATCTGTATCAGTGGCATTGTCTATTGGACCTCCAGTAGTAATTCCATTCTTGCTTTTCGGAGGTTTCTTCTTAAACGCAGGATCCGTTCCTCCCTACTTCGAATGGCTCAGTAATTTGTCCTGGTTCAAATACGGGAACGAAGCCCTACTCATCAACCAATGGGGAGATGTCACTGATATCAAATGCACCAGGATGAACACCACTTGTCCTAAAGACGGACATATCATACTGGAGACTTACAATTTCAGCGAGAGCGATTACCTCATGGACTACTTATGTTTGATTGGACTTATTATTGGATTCAGAACCTTTGCTTTCCTATCTCTACTCTACAGAACATCAGGAAAGAAACACTAG
